# Investigation of gene-by-sex interactions for lipid traits in diverse populations from the population architecture using genomics and epidemiology study

**DOI:** 10.1186/1471-2156-14-33

**Published:** 2013-05-01

**Authors:** Kira C Taylor, Cara L Carty, Logan Dumitrescu, Petra Bůžková, Shelley A Cole, Lucia Hindorff, Fred R Schumacher, Lynne R Wilkens, Ralph V Shohet, P Miguel Quibrera, Karen C Johnson, Brian E Henderson, Jeff Haessler, Nora Franceschini, Charles B Eaton, David J Duggan, Barbara Cochran, Iona Cheng, Chris S Carlson, Kristin Brown-Gentry, Garnet Anderson, Jose Luis Ambite, Christopher Haiman, Loïc Le Marchand, Charles Kooperberg, Dana C Crawford, Steven Buyske, Kari E North, Myriam Fornage

**Affiliations:** 1 Department of Epidemiology, University of North Carolina at Chapel Hill, Chapel Hill, NC, USA; 2 Department of Epidemiology and Population Health, School of Public Health and Information Sciences, University of Louisville, Louisville, KY, USA; 3 Public Health Sciences, Fred Hutchinson Cancer Research Center, Seattle, WA, USA; 4 Center for Human Genetics Research, Vanderbilt University, Nashville, TN, USA; 5 Department of Biostatistics, University of Washington, Seattle, WA, USA; 6 Department of Genetics, Texas Biomedical Research Institute, San Antonio, TX, USA; 7 Office of Population Genomics, National Human Genome Research Institute, National Institutes of Health, Bethesda, MD, USA; 8 Department of Preventive Medicine, Keck School of Medicine, University of Southern California, Los Angeles, CA, USA; 9 Epidemiology Program, University of Hawaii Cancer Center, University of Hawaii, Honolulu, HI, USA; 10 Center of Cardiovascular Research, Department of Medicine, John A. Burns School of Medicine, University of Hawaii, Honolulu, HI, USA; 11 Department of Preventive Medicine, University of Tennessee Health Science Center, Memphis, TN, USA; 12 Zilkha Neurogenetic Institute, University of Southern California, Los Angeles, CA, USA; 13 Department of Family Medicine and Community Health, Alpert Medical School of Brown University, Providence, RI, USA; 14 Integrated Cancer Genomics Division, The Translational Genomics Research Institute, Phoenix, AZ, USA; 15 Sponsored Programs, Baylor College of Medicine, Houston, TX, USA; 16 Cancer Research Center, University of Hawaii, Honolulu, HI, USA; 17 Information Sciences Institute, University of Southern California, Los Angeles, CA, USA; 18 Department of Molecular Physiology and Biophysics, Vanderbilt University, Nashville, TN, USA; 19 Department of Statistics and Biostatistics, Rutgers University, Piscataway, NJ, USA; 20 Carolina Center for Genome Sciences, University of North Carolina at Chapel Hill, Chapel Hill, NC, USA; 21 Division of Epidemiology, Human Genetics, and Environmental Sciences, School of Public Health, University of Texas Health Sciences Center at Houston, Houston, TX, USA; 22 Institute of Molecular Medicine, University of Texas Health Sciences Center at Houston, Houston, TX, USA

**Keywords:** Lipids, Genetics, Cardiovascular disease, Heterogeneity, Sex-specific effect, Association study

## Abstract

**Background:**

High-density lipoprotein cholesterol (HDL-C), low-density lipoprotein cholesterol (LDL-C), and triglyceride (TG) levels are influenced by both genes and the environment. Genome-wide association studies (GWAS) have identified ~100 common genetic variants associated with HDL-C, LDL-C, and/or TG levels, mostly in populations of European descent, but little is known about the modifiers of these associations. Here, we investigated whether GWAS-identified SNPs for lipid traits exhibited heterogeneity by sex in the Population Architecture using Genomics and Epidemiology (PAGE) study.

**Results:**

A sex-stratified meta-analysis was performed for 49 GWAS-identified SNPs for fasting HDL-C, LDL-C, and ln(TG) levels among adults self-identified as European American (25,013). Heterogeneity by sex was established when p_het_ < 0.001. There was evidence for heterogeneity by sex for two SNPs for ln(TG) in the *APOA1/C3/A4/A5/BUD13* gene cluster: rs28927680 (p_het_ = 7.4x10^-7^) and rs3135506 (p_het_ = 4.3x10^-4^), one SNP in *PLTP* for HDL levels (rs7679; p_het_ = 9.9x10^-4^), and one in *HMGCR* for LDL levels (rs12654264; p_het_ = 3.1x10^-5^). We replicated heterogeneity by sex in five of seventeen loci previously reported by genome-wide studies (binomial p = 0.0009). We also present results for other racial/ethnic groups in the supplementary materials, to provide a resource for future meta-analyses.

**Conclusions:**

We provide further evidence for sex-specific effects of SNPs in the *APOA1/C3/A4/A5/BUD13* gene cluster*, PLTP,* and *HMGCR* on fasting triglyceride levels in European Americans from the PAGE study. Our findings emphasize the need for considering context-specific effects when interpreting genetic associations emerging from GWAS, and also highlight the difficulties in replicating interaction effects across studies and across racial/ethnic groups.

## Background

The successes of genome-wide association studies (GWAS) in mapping over 100 loci associated with high-density lipoprotein cholesterol (HDL-C), low-density lipoprotein cholesterol (LDL-C), and triglyceride (TG) levels have advanced our understanding of genomic influences on common diseases [1-11]. However, the translation of such knowledge into clinical and public health applications requires exploration of the epidemiological architecture of these variants. Epidemiologic architecture describes the features of genetic associations in a population-based context that could act as modifiers of these associations [12]. Typical features include demographics (sex, age, and genetic ancestry) and various environmental exposures. The epidemiologic architecture of GWAS-identified genetic variants has largely been unexplored.

Differences in lipid profiles and metabolism between men and women have been well documented [13,14]. Pre-menopausal women have more favorable plasma lipid profiles than men, with lower levels of TG, total cholesterol (TC), and LDL-C, and higher of HDL-C [15-18]. Men and women also differ in lipoprotein particle concentration, subclass distribution, and sizes. For example, women have a larger average size of LDL and HDL particles than men [19,20]. Sex differences in lipid metabolism and lipoprotein kinetics are also well described [21]. However, the molecular basis of sexual dimorphism in lipid metabolism is poorly understood. Differences in sex hormones, body size and composition, and underlying genetic factors may each partially be implicated [17,18,22].

Sex-specific heritabilities of lipid traits have been previously reported [23,24]. Despite considerable advances in the identification of genetic variants influencing plasma lipid levels, few studies have examined the role of sex as a potential modifier of the effects of genetic variation on lipids. Sex-specific genetic associations may provide valuable insight into the factors responsible for the recognized sexual dimorphism in the plasma lipid profile, a major risk factor for cardiovascular disease, and, thus, may have significant public health and clinical relevance [25]. Accounting for the modifying effects of sex in genetic associations of lipid traits may also help in replication of results across studies and in generalization of findings across different populations.

We, therefore, investigated the sex-specific associations of 49 GWAS-identified SNPs with three common lipid traits (LDL-C, HDL-C, and TG) in the diverse cohorts of the Population Architecture using Genomics and Epidemiology (PAGE) study, established in 2008 by the National Human Genome Research Institute to investigate and characterize the epidemiologic architecture of GWAS-identified variants in diverse racial/ethnic groups [12]. We find significant heterogeneity by sex in three previously reported loci, and replicated heterogeneity by sex at nominal levels in three additional loci.

## Results

### Study populations

Table 1 illustrates the diversity of the PAGE study by racial/ethnic group or population, sex, age range, and years of data collection. There were 25,013 European Americans, 10,643 African Americans, 6,134 American Indians, 3,422 Mexican Americans/Hispanics, 827 Japanese/East Asians, and 200 Pacific Islanders/Native Hawaiians. Overall, there were more women (29,330) than men (16,909). Results are presented for the four largest racial/ethnic groups to maintain adequate sample sizes when investigating interactions. HDL-C, LDL-C, and ln(TG), stratified by sex, and racial/ethnic group, are shown in Table 2. Lipid levels are further stratified by cohort in Additional file 1: Table S1. Females had higher mean HDL-C levels than males across all four racial/ethnic groups, though LDL-C and ln(TG) profiles were similar (Table 2).

**Table 1:** Characteristics of PAGE study populations

	EAGLE*	**MEC**	**WHI**	**CALiCo**			
				**ARIC**	**CARDIA**	**CHS**	**SHS**
Type of Study	Cross-sectional	Nested Case Control	Cohort and Clinical Trials	Longitudinal	Longitudinal	Longitudinal	Longitudinal
Focus of Cohort	N/A	Cancer	Women’s Health	Cardiovascular Disease	Cardiovascular Disease	Cardiovascular Disease	Cardiovascular Disease
Years Collected	1991-1994, 1999-2002	1993-1996	1993-1998	1987-2007	1986-2006	1989-1999	1988-present
Median Age	43	67	63	54	25	73	47
Age Range	18-90	48-86	50-79	44-66	18-35	64-96	14-93
% Women	54	36	100	57	56	62	59.3
Sample sizes:							
European Americans	3,909^†^	317	4,688	11,178	2,134	2,787	--
African Americans	1,896	552	1,840	3,770	2,035	550	--
American Indians	--	--	113	--	--	--	6,021
Mexican Americans	2,361	299	762	--	--	--	--

*Abbreviations: Epidemiologic Architecture for Genes Linked to Environment (EAGLE); Multiethnic Cohort (MEC); Women’s Health Initiative (WHI); Causal Variants Across the Life Course (CALiCo); Atherosclerosis Risk in Communities (ARIC); Coronary Artery Risk Development in Young Adults (CARDIA); Cardiovascular Health Study (CHS); Strong Heart Study (SHS).

†The numbers shown are the maximum for any of the three phenotypes (LDL, HDL, or lnTG).

**Table 2:** Mean lipid levels by sex and racial/ethnic group in the PAGE study

**Lipid**	**Sex**	Mean lipid levels (in mg/dL) and cohort ranges*			
		**European Americans**	**African Americans**	**Mexican Americans/Hispanics**	**American Indians**
HDL-C	Male	44.2 (42.6, 49.7)	51.6 (50.4, 53.4)	45.4 (44.9, 48.4)	43.0 (41.9, 43.9)
	Female	57.5 (56.3, 64.3)	57.4 (55.1, 64.2)	52.7 (51.7, 56.0)	48.0 (45.3, 53.1)
LDL-C	Male	131.4 (111.8, 140.1)	124.5 (108.8, 137.8)	124.0 (118.1, 125.0)	109.4 (97.8, 116.6)
	Female	130.7 (105.7, 135.5)	128.6 (111.7, 138.0)	121.4 (117.0, 128.9)	108.4 (99.3, 126.1)
lnTG	Male	4.80 (4.32, 4.88)	4.48 (4.11, 4.63)	4.90 (4.89, 4.95)	4.79 (4.71, 4.85)
	Female	4.79 (4.14, 4.98)	4.51 (4.05, 4.70)	4.94 (4.88, 5.09)	4.82 (4.79, 4.96)

*Ranges are provided for cohort means, not for individuals. For example, for European American males, the cohorts’ mean HDL values ranged from 42.6 (in ARIC) to 49.7 (in MEC). See Additional file 1: Table S1 for detailed information by cohort.

### Heterogeneity by sex in PAGE European Americans

Four SNPs across three different loci showed significant evidence of heterogeneity by sex at our established level of significance (p_het_ < 0.001) in EAs, in HDL-C, ln(TG), and LDL-C levels (Table 3). The complete sex-stratified results for all 49 SNPs in the four largest PAGE racial/ethnic groups are shown in Additional file 1: Tables S2–S4.

**Table 3:** **Loci with evidence of heterogeneity by sex (P**_**het**_ **< 0.001) in PAGE European Americans**

**Gene**	**SNP**	**Coded Allele**	**Pheno-type**	**Males**				**Females**				$P_{\text{comb}}^{†}$	$P_{\text{diff}}^{‡}$	$P_{\text{het}}^{§}$
				**CAF**	**β (SE)**	**P**	**N**	**CAF**	**β (SE)**	**P**	**N**			
*APOA5/BUD13* cluster	rs28927680	C	ln(TG)	0.07	0.13 (0.02)	5.4E-17	9136	0.07	0.04 (0.01)	3.77E-04	15137	2.3E-14	9.8E-19	7.4E-07
	rs3135506	C	ln(TG)	0.06	0.16 (0.02)	1.9E-22	8173	0.06	0.09 (0.01)	1.16E-13	9588	8.4E-32	2.3E-33	4.3E-04
*PLTP*	rs7679	T	HDL	0.82	0.10 (0.30)	7.3E-01	5439	0.82	1.68 (0.37)	6.27E-06	6089	1.9E-03	3.5E-05	9.9E-04
*HMGCR*	rs12654264	A	LDL	0.62	−4.03 (0.54)	8.3E-14	8088	0.62	−1.17 (0.42)	5.98E-03	12424	1.2E-11	1.7E-14	3.1E-05

^*^Abbreviations: CAF = coded allele frequency; SE = standard error.

^†^P-value for SNP-phenotype association in the sex-combined meta-analysis, not allowing for heterogeneity by sex.

^‡^P-value for SNP-phenotype association in the sex-combined meta-analysis, allowing for different effects by sex.

^§^P-value for heterogeneity by sex.

In the *APOA1/C3/A4/A5/BUD13* gene cluster, the C allele of rs28927680 was associated with increased ln(TG), but more strongly in males than females (P_het_ = 7.4×10^-7^)*.* This SNP is in linkage disequilibrium with the other *APOA1/C3/A4/A5* SNP presented in Table 3, rs3135506 (r^2^ = 0.83). In *PLTP*, rs7679 was strongly associated with HDL-C levels in females (p = 6.3×10^-6^), but there was no effect in males (p = 0.73) (p_het_ = 9.9×10^-4^). In *HMGCR*, rs12654264 had a stronger effect on LDL-C levels in males (p = 8.3×10^-14^) than in females (p = 6.0×10^-3^) (p_het_ = 3.1×10^-5^).

The only group with adequate power to detect interactions was EAs, and then only with SNPs that have larger allele frequencies and interaction effect sizes. For SNPs with a minor allele frequency of 0.15, the minimum detectable interaction effects with 80% power for HDL-C were 1.1 mg/dL in EAs, 1.8 mg/dL in AAs, 2.2 mg/dL in AIs, and 2.9 mg/dL in the Mexican population. These numbers represent the minimum detectable difference, comparing the effect size of a SNP on HDL-C levels in males vs. the effect size in females. The mean interaction effect for EAs across all SNPs for HDL-C was 0.35 mg/dL; only two SNPs with MAF > 0.15 had an interaction effect greater than 1.1 mg/dL (Additional file 1: Table S4). Additional file 1: Figure S1 shows the sample sizes required for minimum detectable interactions observable (in standard deviation units) for SNPs of varying allele frequencies.

Although not sufficiently powered to detect interaction in the other ethnic groups we include these results in the supplementary materials as a resource to build sample sizes large enough across these minority groups through meta-analysis. *LDLR* SNP rs6511720 met the criterion for heterogeneity in the Mexican American/Hispanic group (p_het_ = 3.5×10^-4^), with a significant positive effect on ln(TG) levels in males and a significant negative effect in females (Additional file 1: Table S2). The same trend was also observed for American Indians for this SNP. In *HNF4A*, rs1800961 had a significant negative effect on HDL-C levels in AA males but no effect in AA females (p_het_ = 2.6×10^-4^), although the minor allele frequency was only 0.01 (Additional file 1: Table S4). No other SNPs met the pre-specified criterion for declaring significant heterogeneity by sex for any of the three phenotypes in these racial/ethnic groups.

### Replication of previously published reports of heterogeneity

Seventeen SNPs in our dataset have shown evidence of heterogeneity by sex for LDL-C, HDL-C, or TG in previously published genome-wide studies, or are in linkage disequilibrium (R^2^ > 0.2) with previously reported SNPs (Table 4). Heterogeneity by sex was replicated for *PLTP* and the *BUD13* locus at our established level of significance (p < 0.001), as also shown in Table 3. (Note, heterogeneity by sex has been previously reported in *HMGCR* for total cholesterol levels, but we did not have results for total cholesterol and therefore this locus did not meet the criteria for inclusion in Table 4). Heterogeneity by sex was replicated for three additional loci (*LPL, TRIB1,* and *GCKR)* at a nominal p < 0.05 level. The binomial p-value for replicating 5 of 17 findings at p < 0.05 is 0.0009. For all five of these loci, the direction of the interaction was consistent with the previous report [26]. The effect was larger in males for four of the five loci, and stronger in females for one locus.

**Table 4:** Replication of previously reported heterogeneity by sex in PAGE

		**Prior study**					**PAGE**								
**Gene**	**Trait**	**SNP**	**CA**	**CAF**	**Beta (SE), male**	**Beta (SE), female**	**PAGE SNP**	^**§**^**R**^**2**^	**CA**	**CAF**	**Required sample size**^**@**^**for 80% power to detect interaction**	**Beta (SE), male**	**Beta (SE), female**	**P**_**het**_	**Sex with stronger absolute effect (prior, PAGE)**
Significant heterogeneity in PAGE (p_het_ < 0.05)															
*BUD13*	TG	rs11820589	A	0.05	0.131 (0.009)	0.091 (0.008)	rs3135506	0.79	C	0.06	215,337	0.16 (0.02)	0.09 (0.01)	4.3E-4	MM
*GCKR*	TG	rs1260326	T	0.41	0.064 (0.005)	0.050 (0.004)	rs1260326	-	T	0.42	370,777	0.072 (0.009)	0.046 (0.006)	0.013	MM
*LPL*	TG	rs13702	T	0.70	0.070 (0.005)	0.047 (0.004)	rs328	0.39	C	0.90	389,318	−0.110 (0.014)	−0.069 (0.009)	0.013	MM
*PLTP*	HDL	rs6065904	A	0.22	−0.008 (0.003)	−0.031 (0.004)	rs7679	0.86	T	0.82	242,184	0.10 (0.30)	1.68 (0.37)	9.9E-4	FF
*TRIB1*	TG	rs2954033	A	0.30	0.050 (0.005)	0.022 (0.004)	rs2954029	0.36	A	0.54	137,339	−0.059 (0.010)	−0.031 (0.006)	0.015	MM
No significant heterogeneity in PAGE (p_het_ > 0.05)															
*APOB*	TG	rs11902417	A	0.23	−0.038 (0.006)	−0.024 (0.005)	rs693	0.27	T	0.49	>1,000,000	−0.025 (0.009)	−0.014 (0.007)	0.32	MM
*APOB*	LDL	rs531819	T	0.15	−0.092 (0.012)	−0.128 (0.012)	rs562338	0.70	T	0.19	126,763	−5.32 (0.73)	−5.96 (0.66)	0.51	FF
*CETP*	HDL	rs7499892	T	0.17	−0.074 (0.004)	−0.091 (0.005)	rs1864163	0.57	A	0.24	525,658	−2.57 (1.55)	−1.16 (2.68)	0.65	FM
*CILP2*^‡^	LDL	rs10401969	T	0.07	0.15 (0.02)	0.03 (0.01)	rs16996148	1.0	T	0.08	18,530	−3.44 (0.96)	−2.11 (0.76)	0.28	MM
*FADS1*	HDL	rs174548	C	0.70	0.015 (0.003)	0.024 (0.004)	rs174547	0.80	T	0.66	986,476	0.82 (0.22)	0.60 (0.20)	0.47	FM
*GALNT2*	HDL	rs2296065	A	0.85	0.015 (0.004)	0.030 (0.005)	rs2144300	0.26	T	0.60	>1,000,000	0.21 (0.19)	0.63 (0.17)	0.091	FF
*GALNT2*	TG	rs2296065	A	0.85	−0.015 (0.007)	−0.027 (0.005)	rs2144300	0.26	T	0.60	>1,000,000	0.015 (0.008)	0.019 (0.006)	0.73	FF
*LIPC*	HDL	rs2070895	A	0.22	0.033 (0.003)	0.051 (0.004)	rs261332	0.91	A	0.20	375,405	1.77 (0.32)	1.72 (0.42)	0.96	FM
*LIPG*	HDL	rs2156552	A	0.16	−0.023 (0.004)	−0.032 (0.005)	rs2156552	-	T	0.17	>1,000,000	−0.38 (0.24)	−0.56 (0.24)	0.60	FF
*LPL*	HDL	rs10096633	A	0.40	0.014 (0.032)	0.079 (0.011)	rs328	0.87	G	0.10	53,495	1.76 (0.31)	1.93 (0.28)	0.68	FF
*LPL*^‡^	HDL	rs12678919	A	0.12	−0.20 (0.01)	−0.13 (0.01)	rs328	1.0	G	0.10	40,123	1.76 (0.31)	1.93 (0.28)	0.68	MF
*LPL*^†^	HDL	rs2083637	G	0.26	0.149	0.079	rs328	0.43	G	0.10	93,909	1.76 (0.31)	1.93 (0.28)	0.68	MF
*PLTP*	TG	rs6073952	A	0.19	0.009 (0.006)	0.030 (0.005)	rs7679	1.0	T	0.82	247885	0.013 (0.013)	0.009 (0.011)	0.82	FM
*TRIB1*	LDL	rs17321515	A	0.52	0.022 (0.008)	0.045 (0.009)	rs2954029	0.97	A	0.54	63691	1.98 (0.67)	2.16 (0.49)	0.83	FF

^*^Abbreviations: CA = coding allele; CAF = coding allele frequency; SE = standard error; P_het_ = heterogeneity by sex p-value.

All prior SNPs shown are from Asselbergs et al. (2012), except for the three noted.

^†^Aulchenko, Ripatti et al., 2009. Standard errors were not reported for sex-specific effects.

^‡^Teslovich, Musunuru, et al., 2010. Effect sizes were reported for standardized residuals and therefore are not directly comparable to the PAGE results.

^§^LD was calculated using 1000 Genomes Pilot 1 data (CEU population).

^%^Criteria for replication: p_het_ < 0.05 in PAGE and consistent direction of interaction effect (e.g., stronger effects in females in both PAGE and prior study).

^@^Sample size calculations for gene-environment interactions done on Quanto, assuming the CAF from PAGE and the difference in β_f_-β_m_ from the prior study. Calculated sample sizes were then divided by R^2^ (the LD between the PAGE SNP and the prior SNP) to arrive at the final required sample size for 80% power, shown in the table.

We also present the sample size that would have been required in order to have 80% power to replicate the previously reported interaction effect, given the allele frequency in PAGE and the R^2^ between the PAGE SNP and the prior SNP (Table 4). Notably, the required sample sizes to achieve 80% power were >100,000 for 14 of the 17 SNPs. For the one SNP where we were adequately powered to detect interaction (rs10401969 in *CILP2*), we observed a stronger effect in males than in females, consistent with the previous report, though the directions of effect for the T allele appear to be opposite (positive for both males and females in the prior study, but negative for both sexes in ours) [8].

## Discussion

We have examined the sex-specific effects of 49 selected SNPs on circulating lipids in 46,349 PAGE study participants from four racial/ethnic groups. Four SNPs in three loci (*HMGCR, PLTP*, and *APOA5/BUD13*) displayed evidence of sex-SNP interactions for fasting lipid levels according to our pre-specified significance criterion. Heterogeneity by sex for lipid levels had been previously reported in other contexts for each of these loci. We were also able to replicate previously reported heterogeneity by sex at three additional loci (*TRIB1, LPL*, and *GCKR*) at nominal levels of significance, despite being underpowered to detect these interactions, with required sample sizes exceeding 100,000 in most cases.

Although we also analyzed these data in African Americans, American Indians, and Mexican American/Hispanics, we were underpowered to detect interactions in these groups, though two associations reached statistical significance. Sex-SNP interactions that are consistent across populations likely reflect the effects of the biological differences between men and women that are expected to be shared by all population groups. However, differences in power and LD patterns between population groups, as well as possible unrecognized racial/ethnic-specific effects on the sexual dimorphism of lipid levels may have obscured consistent sex-SNP interactions effects across population groups.

Two published GWAS and one gene-centric genome-wide study have tested for heterogeneity by sex for lipid levels among their associated loci. Three of 22 lipid-associated loci (*HMGCR, LPL*, and *NCAN*) exhibited evidence of heterogeneity by sex for either TC or HDL-C levels in a meta-analysis of >20,000 European individuals, though the criterion used for heterogeneity was not stated explicitly [1]. Four of 95 associated loci (*LPL, CILP2, APOE*, and *ZNF664*) exhibited evidence of heterogeneity by sex (P_het_ < 0.0005) for TC, TG, LDL-C, or HDL-C levels in a recent meta-analysis of >100,000 individuals of European ancestry, and seven additional loci were genome-wide significant in one sex but not the other [8]. In the most recent gene-centric genome-wide study, 44 SNPs of ~50,000 were defined as exhibiting heterogeneity by sex for TC, TG, HDL-C, or LDL-C [26].

Association data for seventeen of the previously reported loci from GWAS were available in PAGE. We attempted to replicate the prior findings for TG, HDL and LDL if we had a SNP in linkage disequilibrium (R^2^ > 0.20) with the reported SNP. We did not have association results for total cholesterol, and therefore did not attempt to replicate those findings from any of the prior studies. Five of the previously reported GWAS-identified sex-specific effects consistently replicated in the PAGE study for the same phenotype. There was some overlap in the sample between PAGE and the other meta-analyses; for example PAGE cohorts also analyzed in the gene-centric meta-analysis by Asselbergs et al. include ARIC, WHI, CARDIA, and CHS [26].

Power impacted our ability to detect significant sex effects, with sample sizes necessary for 80% power exceeding 100,000 in 14 of the 17 loci. Although we did not have large sample sizes, we did have the advantage of having previously unreported data for these loci in additional racial/ethnic groups. Of these five loci that were replicated in EAs, the four TG loci (*APO* gene cluster, *GCKR*, *LPL*, and *TRIB1*) had consistent directions of interaction in Mexican Americans/Hispanics, though effects were less consistent for the other racial/ethnic groups (Additional file 1: Table S2). It would be worthwhile to continue this area of investigation to determine whether interaction effects discovered in EAs tend to generalize to other racial/ethnic groups, as this evidence points to generalization in MA/H but not the other groups.

Several candidate gene studies have reported heterogeneity by sex for rs3135506 [27-30], and a nearby SNP was recently reported with heterogeneity by sex (rs11820589; R^2^ = 0.65 with rs28927680) [26]. In PAGE, this variant was associated with ln(TG) in all four major populations tested in the sex-combined meta-analysis [12]. Significant evidence of heterogeneity by sex in PAGE was observed for European Americans and Mexican Americans/Hispanics. In these two groups, significantly stronger effects in males compared with females were observed. A British study reported results consistent with the PAGE study [30]. A Turkish study reported significant associations between triglyceride levels and rs3135506, with a stronger association in women [29]. Sex-differentiated effects have also been reported for triglyceride levels and rs3135506 in a Brazilian population of European descent, again with the female effects stronger than male effects [27]. Klos et al. [28] had previously reported heterogeneity by sex for this SNP in the CARDIA study (data not included in the present study), where it was significantly associated with plasma TG levels in African-American females, but not males. There is also evidence that serum ApoA5 levels are correlated with triglyceride levels and HDL-C levels more strongly in females than in males [31]. Different results observed across different cohorts that represent various genetic ancestries highlight the complexities in replicating and ultimately interpreting sex differences in genetic association studies.

Aulchenko et al. [1] reported heterogeneity by sex for *HMGCR* rs3846662 for total cholesterol. We did not test for heterogeneity by sex for total cholesterol in the PAGE study. We did note, however, that *HMGCR* rs12654264, which is in strong LD with previously reported rs3846662 (r^2^ = 0.87), displayed significant heterogeneity by sex for LDL-C (P_het_ = 3.1×10^-5^), a trait highly correlated with total cholesterol. In the PAGE study, however, the genetic effect was greater in males (β = −4.03) compared with females (β = −1.11), which does not replicate the findings of Aulchenko et al. [1]. The product of the *HMGCR* gene (3-hydroxy-3-methylglutaryl-CoA reductase) is the rate-limiting enzyme of the cholesterol biosynthesis pathway and the target of statins, a class of drugs widely used for the treatment of high cholesterol. Sequence variants of this gene have been associated with variation in response to statin therapy [32]. Among patients with asymptomatic plaques in the carotid artery from the Malmö Diet and Cancer-Cardiovascular Cohort, rs12654264 was associated with reduction in LDL-C levels in response to fluvastatin treatment in men but not women [33].

Heterogeneity by sex for HDL-C levels for *PLTP* (phospholipid transfer protein) has been previously reported, with consistent findings [26]. Studies have provided evidence that PLTP activity may affect HDL particle size [34]. In PAGE, the major allele of rs7679 was associated with higher HDL-C levels in women only.

The locus with the most consistent evidence for heterogeneity by sex across the studies is *LPL*, or lipoprotein lipase. Different SNPs in this gene exhibited heterogeneity by sex for HDL levels in two prior studies, with a larger effect in males [1,8]. In PAGE and in Asselbergs et al., *LPL* exhibited heterogeneity by sex for TG levels, also with a stronger effect in males [26]. LPL (lipoprotein lipase) is the rate-limiting enzyme for hydrolysis of triglycerides in lipoproteins. Polymorphisms and mutations in *LPL* have been associated with lipid metabolism disorders. Hormone levels have been shown to affect regulation of LPL, including thyroid hormone, estrogen, and testosterone [35].

### Strengths and limitations

The diversity of the PAGE study potentially enabled us to examine potential sex-effects across populations, though we were underpowered to detect interactions in three of the four racial/ethnic groups. Physiological, anatomical, or even behavioral differences between men and women that may modify the effects of SNPs on lipid metabolism are expected to be largely shared across racial/ethnic groups. Hence, the consistent effects across racial/ethnic groups described above provide added support to the findings reported here.

Some limitations must be acknowledged, including sample size. Power to detect interaction effects typically requires substantially larger samples than those for main effects [36,37]. Sample size was greatest for European-Americans and, not unexpectedly, most evidence of sex-specific effects in this study was observed in this population. The required sample sizes to detect interaction for these loci (generally exceeding 100,000, and sometimes exceeding 1,000,000) should alert other investigators to the difficulty of replicating interaction effects.

The PAGE study cohorts differ in many aspects, including study design, period of collection, demographics and cardiovascular and metabolic risk factors of the participants. These differences may have further reduced our power to detect significant modifying effects of sex on SNPs-lipids associations. Indeed, sex differences not only represent biological differences between men and women but also encompass or are confounded by social and behavioral differences between the two sexes. Difficulties in assessing and accounting for such factors in a consistent and accurate manner across the multiple cohorts likely further reduced our power to detect interaction with genetic factors.

This difficulty of replicating heterogeneity by sex is compounded by the nature of the tested SNPs, which are likely to be in LD with the causal variant(s) but not themselves causal. Differences in LD patterns across studies and racial/ethnic groups may have hampered our ability to detect consistent sex modification effects in some population groups.

Claims of modification by sex have been difficult to replicate for most complex diseases and many studies lack the proper documentation for the claim of significant sex-effects [38]. In this study, we note that the three loci which met *our a priori* criterion for significant interaction had been previously reported to display heterogeneity by sex, suggesting that the initial reports for these loci were not attributable to type I error.

Differences in sex hormone levels have been hypothesized to play a role in the sexual dimorphism of circulating lipids. In this study, we did not examine whether menopausal status modified the association of these SNPs with lipid levels in women. Future such investigations may help shed light on the biological basis of the sex-specific associations reported here.

## Conclusions

Using a rigorous methodology and the diverse populations of the PAGE study, we have confirmed previously reported heterogeneity by sex for lipid levels for six loci. Genotype-sex interactions may represent an important source of genetic variation that may contribute to the “missing heritability” of complex traits. Although challenging, assessment of sex-specific associations should be more widely considered in order to characterize the genetic architecture of complex, sexually dimorphic traits, such as lipids.

## Methods

### Study populations and phenotypes

The study population included 46,239 individuals from cohorts which are part of the PAGE study, a collaborative program across four large population-based studies or consortia, including EAGLE (Epidemiologic Architecture for Genes Linked to Environment), based on three National Health and Nutrition Examination Surveys (NHANES) [39]; the Multiethnic Cohort (MEC) [40]; the Women’s Health Initiative (WHI) [41]; and the Causal variants Across the Life Course (CALiCo) consortium, which encompasses five studies: Atherosclerosis Risk in Communities (ARIC) [42], Coronary Artery Risk in Young Adults (CARDIA) [43], the Cardiovascular Health Study (CHS) [44], Strong Heart Family Study (SHFS) and Strong Heart Study (SHS) [45]. Details about the design of the PAGE study have been previously published [12]. All participants were consented and all studies were approved by Institutional Review Boards at their respective sites.

Serum HDL-C, triglycerides, and total cholesterol were measured using standard enzymatic methods. LDL-C was calculated using the Friedewald equation, with missing values assigned for samples with triglyceride levels greater than 400 mg/dl. For PAGE cohorts with longitudinal data, measurements from the baseline examination were used in the analyses.

The PAGE study participants who were less than 18 years of age and those fasting for less than 8 hours prior to the blood draw were excluded from analyses. Participants with triglycerides values >1,000 mg/dl were excluded from analyses of that trait. The distribution of triglyceride levels was skewed and thus values were natural log transformed prior to analysis. A further description of the study design, methods of data collection and participants’ characteristics for each of the cohorts have been presented elsewhere [46].

### SNP selection and genotyping

Detailed methods of SNP selection and genotyping have been described previously [12]. Briefly, a total of 52 SNPs previously associated with HDL-C, LDL-C, and/or triglycerides in published candidate gene and genome-wide association studies (through 2008) were targeted for genotyping in two or more PAGE cohorts. Of these, three (*CETP* rs1800775, *APOE* rs429358, and *APOE* rs7412) failed at all PAGE study sites that attempted genotyping; therefore, a total of 49 SNPs were included in this analysis. Genotyping was performed by each of the four PAGE studies using commercially available genotyping arrays (Affymetrix 6.0, Illumina 370CNV BeadChip), custom mid- and low-throughput assays (TaqMan, Sequenom, Illumina GoldenGate on the BeadXpress), or a combination thereof. Quality control was implemented at each study site independently. Only SNPs with high call rates (>95%) were included in the analyses. In addition, all PAGE study sites genotyped 360 DNA samples from the International HapMap Project for concordance analysis.

### Statistical methods

#### Cohort-specific analyses

Statistical analyses were performed separately by each cohort following the same analysis plan. Within each racial/ethnic group and sex stratum, linear regression was used to evaluate the association of each SNP with HDL-C, LDL-C, or natural log-transformed TG levels, assuming an additive genetic model. Models were adjusted for age and field center (for multi-center studies). Previous PAGE study results for lipids demonstrated that further adjustment for body mass index, current smoking, type 2 diabetes, post-menopausal status, current hormone use, myocardial infarction, and ancestry using principal components, did not meaningfully impact the association of these SNPs with lipid levels [46]. Analyses were performed without regard to lipid lowering medication status given that relatively few participants (<10%) reported such medication use, and their inclusion did not appreciably alter results in previous PAGE lipids work [46].

#### Meta-analyses

Sex-specific beta coefficients were combined within each racial/ethnic group using an inverse variance-weighted fixed-effects meta-analysis, using METAL software [47]. For each SNP, significance of association within each stratum was evaluated using a 1-df Chi-square test. Heterogeneity of effects between the sexes was then evaluated using the following 1-df Chi-square test: (β_m_-β_f_)/(SE_m_^2^ + SE_f_^2^) ~ χ^2^_1_, where β_m_ and β_f_ represent the meta-analyzed effect estimates among males and females, respectively. In addition, for each SNP, sex-differentiated tests of association were performed using a Chi-square test (2 df) as described by Magi et al. [48] in which the 1-df Chi-square statistics for the SNP-phenotype associations for males and females are summed. This method yields a single P-value of association but permits the effect estimates for males and females to differ. It also permits inclusion of single-sex studies such as WHI that otherwise would not be able to contribute to a traditional interaction meta-analysis. Reported P-values were not adjusted for multiple testing.

Reported P-values include P_comb_, the genotype-phenotype association p-value not allowing for a sex-specific association; P_diff,_ the genotype-phenotype association P-value allowing for different effects by sex; and P_het_, the P-value for heterogeneity of sex effects. The *a priori* criterion for heterogeneity was set at P_het_ < 0.001 (a Bonferroni correction for the number of SNPs examined; 0.05/49).

#### Sample size and power calculations

Sample size and power for gene-environment interactions were calculated using Quanto software (http://hydra.usc.edu/gxe/). To calculate sample sizes required to replicate previous findings, we used the following assumptions: power = 80%; alpha = 0.05 (two-sided); interaction effect size = (male effect – female effect, in standard deviation units); minor allele frequency = the minor allele frequency in the PAGE population; additive genetic model. To calculate minimum detectable effect sizes, we set sample size equal to the PAGE sample size but kept the other assumptions. Because none of the other racial/ethnic groups in PAGE had adequate power to detect gene-environment interactions, only the European-American results are presented in the main text. Results for the other groups are presented in the supplementary materials.

## Abbreviations

AA: African-American; AI: American Indian; ARIC: Atherosclerosis Risk In Communities; CAF: Coded allele frequency; CALICO: Causal Variants Across the Life Course; CARDIA: Coronary Artery Risk Development in Young Adults; CHS: Cardiovascular Health Study; df: Degrees of freedom; EA: European-American; EAGLE: Epidemiologic Architecture for Genes Linked to Environment; GWAS: Genome-wide association study; HDL-C: High density lipoprotein-cholesterol; LDL-C: Low density lipoprotein-cholesterol; MA/H: Mexican American/Hispanic; MEC: Multi-Ethnic Cohort; PAGE: Population Architecture using Genomics and Epidemiology; Pcomb: P-value for association, not allowing for different effects by sex; Pdiff: P-value for association, allowing different effect estimates for males and females; Phet: P-value for heterogeneity; R/E: Race/Ethnicity; TC: Total cholesterol; TG: Triglycerides; SE: Standard error; SHS: Strong Heart Study; SNP: Single Nucleotide Polymorphism; WHI: Women’s Health Initiative.

## Competing interests

The authors declare that they have no competing interests.

## Authors’ contributions

KCT and MF designed the meta-analysis, conducted the meta-analysis and power analysis, and drafted and edited the manuscript. CLC, LD, PB, SAC, LH, and FRS assisted with planning the study, and interpretation of results, and with drafting and editing the manuscript. LRW, RVS, PMQ, KCJ, BEH, JH, NF, CBE, DJD, BC, and IC conducted the single-study analyses in the original cohorts and participated in discussions regarding the manuscript. CSC, KBG, GA, JLA, CH, LLM, and CK oversaw the single-study analyses and participated in discussions regarding the manuscript. DCC, SB, and KEN assisted with the design of the meta-analysis, and assisted with the drafting and editing the manuscript. All authors have read and approved the final manuscript.

## Supplementary Material

Additional file 1 This file contains Supplementary Tables 1-4 and Supplementary Figure 1 as described in the text. Click here for file
