# Supplementary material for: Investigation of gene-by-sex interactions for lipid traits in diverse populations from the population architecture using genomics and epidemiology study
Source: BMC Genet. 2013 May 1;14:33. doi: 10.1186/1471-2156-14-33 (PMC3669109; doi:10.1186/1471-2156-14-33)

Supplementary Materials

Supplementary Table 1. Mean lipid levels by sex and cohort in the PAGE studies.

Study	Racial/ethnic group or population*	HDL mean (SD*) in mg/dL		LDL mean (SD) in mg/dL		ln(TG) mean (SD) in mg/dL	
		Males	Females	Males	Females	Males	Females
EAGLE	EA	45.2 (12.5)	56.5 (16.4)	126.6 (35.3)	127.1 (36.8)	4.88 (0.54)	4.80 (0.53)
	AA	51.5 (16.0)	56.1 (17.2)	123.1 (38.1)	121.6 (39.3)	4.57 (0.53)	4.50 (0.48)
	MA/H	44.9 (12.3)	51.7 (13.4)	125.0 (34.2)	117.0 (32.4)	4.89 (0.58)	4.88 (0.53)
MEC	EA	49.7 (13.1)	64.3 (16.8)	112.3 (31.9)	126.0 (34.5)	4.65 (0.52)	4.81 (0.47)
	AA	51.5 (13.8)	64.2 (15.1)	123.9 (44.0)	126.1 (39.3)	4.63 (0.46)	4.56 (0.44)
	MA/H	48.4 (11.5)	56.0 (14.5)	118.1 (36.4)	128.9 (37.5)	4.95 (0.50)	5.09 (0.43)
WHI	EA	--	58.3 (15.5)	--	132.2 (34.5)	--	4.98 (0.45)
	AA	--	57.4 (15.1)	--	134.1 (39.2)	--	4.70 (0.40)
	AI	--	53.1 (13.7)	--	126.1 (37.8)	--	4.96 (0.49)
	MA/H	--	53.9 (14.5)	--	127.6 (35.0)	--	5.04 (0.45)

ARIC	EA	42.6 (12.4)	57.4 (17.0)	140.1 (35.5)	135.5 (39.5)	4.86 (0.52)	4.73 (0.50)
	AA	50.4 (16.8)	58.2 (17.4)	137.8 (41.9)	138.0 (44.0)	4.63 (0.50)	4.58 (0.44)
CARDIA	EA	47.2 (11.0)	56.3 (13.0)	111.8 (30.9)	105.7 (28.7)	4.32 (0.54)	4.14 (0.46)
	AA	53.4 (13.4)	55.1 (12.7)	108.8 (32.5)	111.7 (31.3)	4.11 (0.48)	4.05 (0.43)
CHS	EA	46.0 (11.0)	57.3 (14.4)	121.6 (31.7)	130.8 (33.9)	4.84 (0.50)	4.91 (0.48)
	AA	51.9 (13.4)	60.7 (14.9)	122.1 (32.8)	133.8 (37.7)	4.62 (0.47)	4.65 (0.42)
SHS	AI – Arizona	43.2 (14.0)	45.3 (11.3)	97.8 (29.3)	99.3 (29.5)	4.80 (0.56)	4.86 (0.52)
	AI – South Dakota	43.9 (14.2)	48.7 (14.2)	116.6 (32.4)	114.6 (32.2)	4.71 (0.66)	4.79 (0.57)
	AI – Oklahoma	41.9 (12.2)	49.4 (13.7)	113.8 (31.4)	109.7 (30.9)	4.85 (0.52)	4.81 (0.55)

*Abbreviations: EA = European American; AA= African American; MA/H = Mexican American/Hispanic; AI = American Indian; SD=standard deviation

Supplementary Table 2. Sex-specific associations of GWAS-identified lipid SNPs with ln(TG) levels in the PAGE study.

			Males				Females						
SNP/Gene	Coded Allele	R/E[*]	CAF	β (SE)	P	N	CAF	β (SE)	P	N	P_{comb}[†]	P_{diff}[‡]	P_{het}[§]
rs1883025	A	EA	0.25	0.00 (0.01)	8.65E-01	5877	0.25	-0.02 (0.01)	6.92E-03	6804	3.31E-02	2.56E-02	9.52E-02
ABCA1		AA	0.34	-0.02 (0.03)	4.39E-01	686	0.34	0.00 (0.01)	7.79E-01	890	9.81E-01	7.13E-01	4.11E-01
		AI	--[||]	--	--	--	--	--	--	--	--	--	--
		MA/H	0.28	0.01 (0.03)	7.88E-01	961	0.26	0.01 (0.01)	3.90E-01	978	3.73E-01	6.67E-01	8.99E-01
rs3890182	A	EA	0.12	-0.01 (0.01)	3.11E-01	8419	0.12	-0.01 (0.01)	1.91E-01	14302	9.93E-02	2.55E-01	8.93E-01
ABCA1		AA	0.12	0.01 (0.02)	6.53E-01	3213	0.12	-0.02 (0.01)	4.21E-02	5883	1.24E-01	1.15E-01	1.61E-01
		AI	0.04	0.01 (0.04)	7.90E-01	2613	0.04	-0.04 (0.03)	2.14E-01	4219	3.89E-01	4.46E-01	3.50E-01
		MA/H	0.08	-0.05 (0.04)	2.41E-01	1129	0.09	-0.03 (0.02)	6.93E-02	1815	3.34E-02	9.67E-02	7.05E-01
rs4149268	A	EA	0.37	0.01 (0.01)	5.66E-01	5866	0.37	-0.02 (0.01)	1.50E-02	6650	9.46E-02	4.39E-02	6.29E-02
ABCA1		AA	0.67	0.00 (0.02)	9.94E-01	2093	0.68	0.00 (0.01)	9.64E-01	2725	9.72E-01	9.99E-01	9.77E-01
		AI	--	--	--	--	--	--	--	--	--	--	--
		MA/H	0.31	-0.01 (0.03)	5.80E-01	1082	0.32	0.00 (0.01)	8.01E-01	1000	6.36E-01	8.31E-01	7.03E-01
rs6544713	T	EA	0.31	0.02 (0.01)	7.96E-02	6133	0.32	0.00 (0.01)	9.10E-01	11285	2.50E-01	2.14E-01	1.84E-01
ABCG8		AA	0.17	-0.01 (0.02)	6.61E-01	2824	0.17	0.02 (0.01)	5.30E-02	5433	1.69E-01	1.40E-01	1.53E-01
		AI	0.09	-0.03 (0.03)	3.05E-01	2609	0.09	0.00 (0.02)	8.30E-01	4213	4.32E-01	5.78E-01	4.88E-01
		MA/H	0.16	-0.04 (0.07)	5.89E-01	182	0.22	-0.01 (0.03)	7.60E-01	851	6.29E-01	8.25E-01	6.97E-01
rs1748195	C	EA	0.67	0.05 (0.01)	1.12E-05	5705	0.66	0.02 (0.01)	6.54E-04	10969	2.00E-07	1.91E-07	4.93E-02
ANGPLT3		AA	0.34	0.01 (0.02)	5.45E-01	1739	0.35	0.01 (0.01)	1.49E-01	4156	1.18E-01	2.94E-01	9.69E-01
		AI	--	--	--	--	0.61	0.16 (0.07)	2.45E-02	132	2.45E-02		
		MA/H	0.60	0.05 (0.03)	4.10E-02	959	0.59	0.02 (0.01)	2.17E-02	1746	3.75E-03	8.88E-03	3.08E-01
rs2967605	A	EA	0.18	0.02 (0.01)	4.35E-02	8417	0.18	0.02 (0.01)	1.39E-02	9863	1.51E-03	6.32E-03	8.17E-01
ANGPLT4		AA	0.21	0.00 (0.01)	9.33E-01	3218	0.21	0.00 (0.01)	8.79E-01	4371	8.62E-01	9.85E-01	9.93E-01
		AI	0.30	0.02 (0.02)	3.01E-01	2609	0.29	0.00 (0.01)	9.54E-01	4077	5.09E-01	5.85E-01	4.26E-01
		MA/H	0.23	-0.04 (0.03)	1.80E-01	1137	0.24	0.03 (0.01)	1.97E-02	1052	1.30E-01	2.68E-02	2.61E-02
rs28927680	G	EA	0.93	-0.13 (0.02)	5.44E-17	9136	0.93	-0.04 (0.01)	3.77E-04	15137	2.25E-14	9.81E-19	7.42E-07
APOA1/C3/		AA	0.84	0.03 (0.02)	9.08E-02	3507	0.84	-0.01 (0.01)	4.98E-01	6340	7.98E-01	1.90E-01	7.13E-02
A4/A5/BUD13		AI	0.87	-0.12 (0.02)	1.86E-07	2612	0.87	-0.11 (0.02)	4.85E-12	4220	5.14E-18	5.08E-17	7.47E-01
		MA/H	0.85	0.07 (0.03)	2.63E-02	1142	0.87	0.02 (0.01)	1.79E-01	1830	3.30E-02	3.43E-02	1.38E-01
rs3135506	C	EA	0.06	0.16 (0.02)	1.88E-22	8173	0.06	0.09 (0.01)	1.16E-13	9588	8.38E-32	2.28E-33	4.25E-04
APOA5		AA	0.06	0.09 (0.03)	2.32E-03	2672	0.06	0.09 (0.02)	1.90E-08	3631	1.68E-10	1.30E-09	8.15E-01
		AI	0.12	0.13 (0.02)	8.96E-08	2621	0.13	0.12 (0.02)	3.01E-13	4090	1.53E-19	1.63E-18	8.74E-01

		MA/H	0.14	0.15 (0.03)	1.38E-05	1130	0.13	0.06 (0.02)	1.09E-03	1045	1.18E-06	3.76E-07	1.48E-02
rs964184	C	EA	0.86	-0.17 (0.02)	5.87E-26	4180	0.86	-0.13 (0.01)	1.28E-25	9117	6.30E-49	1.14E-48	3.52E-02
APOA1/C3/		AA	0.80	0.02 (0.03)	4.01E-01	1033	0.80	-0.04 (0.01)	9.15E-03	3230	5.07E-02	2.35E-02	5.50E-02
A4/A5		AI	--	--	--	--	0.78	-0.17 (0.07)	1.43E-02	132	1.43E-02	--	--
		MA/H	--	--	--	--	0.75	-0.12 (0.03)	1.66E-05	772	1.66E-05	--	--
rs562338	T	EA	0.19	-0.01 (0.01)	3.84E-01	6985	0.19	-0.01 (0.01)	5.12E-01	12576	2.92E-01	5.52E-01	7.76E-01
APOB		AA	0.59	-0.02 (0.01)	8.97E-02	2527	0.59	-0.02 (0.01)	4.35E-02	4986	8.93E-03	3.08E-02	7.33E-01
		AI	0.08	-0.03 (0.03)	3.65E-01	2616	0.08	-0.02 (0.02)	2.92E-01	4216	1.66E-01	3.81E-01	9.13E-01
		MA/H	0.16	-0.14 (0.07)	4.08E-02	182	0.19	-0.04 (0.03)	1.66E-01	853	3.67E-02	4.71E-02	1.87E-01
rs693	T	EA	0.49	0.02 (0.01)	5.50E-03	6538	0.49	0.01 (0.01)	4.63E-02	12180	1.07E-03	2.90E-03	3.24E-01
APOB		AA	0.24	0.02 (0.02)	1.72E-01	2463	0.23	0.02 (0.01)	3.06E-02	4878	1.06E-02	3.80E-02	9.86E-01
		AI	0.33	0.01 (0.02)	7.06E-01	2614	0.33	0.02 (0.01)	1.57E-01	4207	1.71E-01	3.43E-01	6.07E-01
		MA/H	0.37	0.06 (0.05)	2.34E-01	182	0.41	0.02 (0.02)	3.62E-01	852	1.92E-01	3.26E-01	4.62E-01
rs754523	T	EA	0.68	-0.01 (0.01)	5.98E-01	6541	0.68	-0.01 (0.01)	4.29E-01	7770	3.46E-01	6.37E-01	9.11E-01
APOB		AA	0.78	0.00 (0.02)	8.15E-01	2457	0.79	-0.01 (0.01)	5.23E-01	3353	7.16E-01	7.93E-01	5.65E-01
		AI	0.69	-0.02 (0.02)	2.80E-01	2612	0.68	-0.01 (0.01)	4.88E-01	4075	2.32E-01	4.38E-01	6.38E-01
		MA/H	0.71	-0.10 (0.06)	8.37E-02	180	0.69	-0.06 (0.08)	4.75E-01	80	6.82E-02	1.74E-01	6.75E-01
rs10402271	T	EA	0.67	0.00 (0.01)	9.09E-01	5544	0.67	-0.01 (0.01)	4.71E-01	6631	5.36E-01	7.66E-01	6.99E-01
APOE/C1/		AA	0.84	0.00 (0.02)	8.32E-01	1621	0.84	-0.04 (0.02)	2.00E-02	2176	8.34E-02	6.54E-02	1.17E-01
C4		AI	--	--	--	--	--	--	--	--	--	--	--
		MA/H	0.61	0.05 (0.06)	3.90E-01	183	0.63	0.03 (0.07)	6.39E-01	81	3.35E-01	6.19E-01	8.58E-01
rs4420638	A	EA	0.82	-0.05 (0.01)	2.06E-05	7246	0.83	-0.03 (0.01)	5.24E-04	13031	1.65E-07	2.77E-07	9.74E-02
APOE/C1/		AA	0.79	0.10 (0.02)	3.17E-07	2009	0.80	0.03 (0.01)	4.53E-04	4637	7.17E-08	4.38E-09	2.14E-03
C4		AI	0.92	-0.03 (0.03)	3.20E-01	2619	0.92	-0.03 (0.02)	1.55E-01	4214	8.30E-02	2.22E-01	9.76E-01
		MA/H	0.90	-0.11 (0.04)	1.69E-02	905	0.89	0.00 (0.02)	9.63E-01	1689	3.44E-01	5.75E-02	2.83E-02
rs4803750	A	EA	0.93	-0.04 (0.02)	6.58E-02	5566	0.93	-0.01 (0.02)	5.64E-01	6650	1.04E-01	1.56E-01	2.98E-01
BCL3		AA	0.92	0.04 (0.03)	2.62E-01	1625	0.92	-0.01 (0.02)	5.51E-01	2193	8.57E-01	4.46E-01	2.08E-01
		AI	--	--	--	--	--	--	--	--	--	--	--
		MA/H	0.86	0.05 (0.08)	5.54E-01	183	0.84	0.13 (0.10)	2.18E-01	81	2.25E-01	3.94E-01	5.31E-01
rs646776	A	EA	0.78	0.01 (0.01)	4.32E-01	6709	0.79	0.00 (0.01)	9.00E-01	7982	6.84E-01	7.28E-01	4.94E-01
CELSR2/		AA	0.64	-0.03 (0.01)	3.91E-02	2753	0.65	0.00 (0.01)	9.89E-01	3799	2.13E-01	1.19E-01	1.00E-01
PSCR1/		AI	0.80	0.00 (0.03)	9.47E-01	1072	0.80	0.02 (0.02)	5.05E-01	1743	5.70E-01	7.99E-01	7.22E-01
SORT1		MA/H	0.80	-0.04 (0.07)	5.39E-01	182	0.74	-0.05 (0.08)	5.29E-01	80	3.82E-01	6.79E-01	9.22E-01
rs599839	A	EA	0.77	0.01 (0.01)	1.64E-01	6992	0.77	0.00 (0.01)	9.33E-01	8155	4.05E-01	3.78E-01	2.63E-01
CELSR2/		AA	0.28	0.01 (0.02)	6.31E-01	2527	0.28	0.01 (0.01)	5.83E-01	3471	4.66E-01	7.66E-01	9.65E-01
PSCR1/		AI	0.78	0.03 (0.02)	1.03E-01	2593	0.78	0.02 (0.01)	2.30E-01	4068	5.22E-02	1.29E-01	5.64E-01

SORT1		MA/H	0.77	-0.01 (0.06)	8.35E-01	178	0.72	0.00 (0.08)	9.98E-01	81	8.69E-01	9.78E-01	8.98E-01
rs12596776	C	EA	0.90	0.01 (0.02)	7.35E-01	5856	0.90	-0.01 (0.01)	5.12E-01	6668	7.23E-01	7.62E-01	5.18E-01
CETP		AA	0.94	-0.03 (0.03)	3.52E-01	2100	0.94	0.01 (0.02)	4.78E-01	2716	8.83E-01	5.04E-01	2.45E-01
		AI	--	--	--	--	--	--	--	--	--	--	--
		MA/H	0.94	-0.04 (0.05)	4.47E-01	1131	0.94	0.01 (0.02)	6.61E-01	1057	9.70E-01	6.80E-01	3.80E-01
rs1566439	A	EA	0.60	0.03 (0.01)	2.41E-03	6988	0.60	0.00 (0.01)	8.65E-01	8386	6.51E-02	9.84E-03	1.57E-02
CETP		AA	0.78	-0.03 (0.02)	1.26E-01	2309	0.78	0.00 (0.01)	7.98E-01	3081	3.16E-01	3.01E-01	2.37E-01
		AI	--	--	--	--	--	--	--	--	--	--	--
		MA/H	0.54	0.02 (0.02)	5.15E-01	1135	0.54	-0.01 (0.01)	3.02E-01	1058	5.23E-01	4.75E-01	2.98E-01
rs1864163	A	EA	0.24	0.07 (0.06)	2.36E-01	182	0.21	-0.08 (0.08)	3.01E-01	109	7.70E-01	2.90E-01	1.22E-01
CETP		AA	0.27	0.03 (0.04)	3.79E-01	367	0.24	-0.01 (0.08)	8.96E-01	84	4.61E-01	6.74E-01	6.20E-01
		AI	--	--	--	--	--	--	--	--	--	--	--
		MA/H	0.27	-0.02 (0.06)	6.88E-01	183	0.30	0.00 (0.09)	9.86E-01	82	7.29E-01	9.22E-01	8.38E-01
rs3764261	T	EA	0.32	-0.02 (0.01)	8.42E-03	8587	0.33	-0.01 (0.01)	1.50E-01	10218	6.88E-03	1.10E-02	1.91E-01
CETP		AA	0.31	-0.01 (0.01)	2.67E-01	3403	0.32	-0.01 (0.01)	2.46E-01	4641	1.16E-01	2.75E-01	7.43E-01
		AI	0.31	-0.03 (0.02)	1.23E-01	2597	0.31	-0.04 (0.01)	5.19E-04	4061	2.06E-04	7.33E-04	4.23E-01
		MA/H	0.34	-0.02 (0.03)	3.58E-01	1083	0.32	-0.03 (0.01)	3.68E-02	1001	2.27E-02	7.40E-02	9.17E-01
rs9989419	A	EA	0.40	0.01 (0.01)	1.21E-01	7706	0.40	0.01 (0.01)	2.66E-01	8959	6.38E-02	1.62E-01	6.51E-01
CETP		AA	0.59	-0.02 (0.01)	1.50E-01	2812	0.60	0.01 (0.01)	4.91E-01	3902	7.57E-01	2.80E-01	1.18E-01
		AI	0.26	0.00 (0.02)	8.66E-01	2609	0.25	0.02 (0.01)	2.53E-01	4074	3.09E-01	5.13E-01	5.85E-01
		MA/H	0.34	0.02 (0.05)	6.46E-01	181	0.40	-0.06 (0.08)	4.67E-01	81	9.67E-01	6.91E-01	3.90E-01
rs16996148	T	EA	0.08	-0.04 (0.01)	2.96E-03	8377	0.08	-0.02 (0.01)	1.20E-02	14242	1.86E-04	5.12E-04	2.78E-01
CILP2/		AA	0.15	0.00 (0.02)	7.72E-01	3190	0.15	0.00 (0.01)	8.81E-01	5858	9.91E-01	9.48E-01	7.44E-01
PBX4/		AI	0.04	-0.06 (0.04)	1.35E-01	2618	0.04	-0.06 (0.03)	3.12E-02	4219	8.76E-03	3.21E-02	9.71E-01
NCAN1		MA/H	0.06	-0.04 (0.05)	4.34E-01	1141	0.06	-0.05 (0.02)	2.33E-02	1832	1.69E-02	5.62E-02	8.28E-01
rs174547	T	EA	0.66	-0.05 (0.01)	1.17E-06	6796	0.66	-0.03 (0.01)	5.44E-05	7721	1.26E-09	2.10E-09	8.20E-02
FADS1		AA	0.91	-0.05 (0.02)	3.56E-02	3186	0.92	-0.04 (0.01)	3.61E-03	4324	3.42E-04	1.59E-03	8.18E-01
		AI	0.17	-0.08 (0.02)	4.19E-04	2211	0.15	-0.07 (0.02)	1.29E-04	3517	2.12E-07	1.28E-06	6.72E-01
		MA/H	0.39	-0.03 (0.02)	1.55E-01	1080	0.40	-0.04 (0.01)	2.71E-03	1003	9.04E-04	4.03E-03	9.68E-01
rs2144300	T	EA	0.60	-0.02 (0.01)	5.97E-02	8327	0.60	-0.02 (0.01)	6.08E-04	14120	9.83E-05	4.74E-04	7.28E-01
GALNT2		AA	0.14	0.00 (0.02)	8.41E-01	3200	0.16	-0.01 (0.01)	2.67E-01	5884	2.83E-01	5.30E-01	7.31E-01
		AI	0.55	-0.01 (0.02)	7.30E-01	2607	0.55	-0.01 (0.01)	3.03E-01	4205	3.00E-01	5.54E-01	7.44E-01
		MA/H	0.57	-0.01 (0.02)	6.65E-01	1142	0.55	-0.02 (0.01)	3.73E-02	1829	3.72E-02	1.04E-01	6.68E-01
rs1260326	T	EA	0.42	0.07 (0.01)	3.30E-16	6964	0.42	0.05 (0.01)	8.01E-16	12896	4.25E-29	2.55E-29	1.30E-02
GCKR		AA	0.17	0.00 (0.03)	9.66E-01	882	0.15	0.04 (0.01)	8.29E-04	2748	1.80E-03	3.72E-03	2.31E-01
		AI	--	--	--	--	0.29	0.15 (0.09)	8.52E-02	131	8.52E-02	--	--

		MA/H	0.32	0.06 (0.03)	2.42E-02	947	0.33	0.03 (0.01)	5.38E-03	1737	6.04E-04	1.64E-03	3.04E-01
rs780094	A	EA	0.40	0.07 (0.01)	2.10E-14	6544	0.40	0.06 (0.01)	5.60E-16	7779	9.18E-29	1.08E-27	6.46E-01
GCKR		AA	0.19	0.01 (0.02)	4.46E-01	2461	0.18	0.03 (0.01)	1.49E-02	3359	1.68E-02	3.85E-02	3.71E-01
		AI	0.21	0.07 (0.02)	3.61E-04	2615	0.20	0.03 (0.01)	7.63E-02	4086	3.94E-04	3.56E-04	6.90E-02
		MA/H	0.34	0.12 (0.06)	4.68E-02	182	0.33	0.03 (0.08)	6.85E-01	81	6.46E-02	1.28E-01	4.02E-01
rs12654264	A	EA	0.62	0.02 (0.01)	3.09E-02	8415	0.62	0.00 (0.01)	6.92E-01	14295	1.26E-01	8.99E-02	1.15E-01
HMGCR		AA	0.67	0.03 (0.01)	3.22E-02	3209	0.67	0.00 (0.01)	5.16E-01	5874	6.10E-01	8.18E-02	2.94E-02
		AI	0.57	-0.04 (0.02)	2.99E-02	2610	0.59	0.00 (0.01)	7.02E-01	4219	1.14E-01	8.78E-02	1.24E-01
		MA/H	0.61	-0.02 (0.03)	4.18E-01	1129	0.62	0.00 (0.01)	8.13E-01	1814	9.15E-01	7.01E-01	4.03E-01
rs2650000	T	EA	0.35	-0.01 (0.01)	1.58E-01	7932	0.35	0.00 (0.01)	8.31E-01	9459	5.23E-01	3.61E-01	2.02E-01
HNF1A		AA	0.12	0.04 (0.02)	3.95E-02	3134	0.12	-0.01 (0.01)	3.87E-01	4250	7.13E-01	8.25E-02	2.76E-02
		AI	0.41	0.00 (0.02)	8.70E-01	2611	0.41	0.01 (0.01)	5.49E-01	4087	5.61E-01	8.24E-01	8.25E-01
		MA/H	0.36	0.02 (0.02)	5.37E-01	1137	0.36	0.01 (0.01)	3.21E-01	1050	2.44E-01	5.04E-01	9.06E-01
rs1800961	T	EA	0.03	-0.05 (0.02)	5.32E-02	7916	0.03	-0.03 (0.02)	4.40E-02	9451	5.97E-03	2.03E-02	6.31E-01
HNF4A		AA	0.01	-0.06 (0.07)	3.50E-01	3127	0.01	0.00 (0.05)	9.84E-01	4245	5.99E-01	6.46E-01	4.40E-01
		AI	0.03	-0.01 (0.04)	8.93E-01	2617	0.03	0.01 (0.03)	8.43E-01	4089	9.43E-01	9.72E-01	8.19E-01
		MA/H	0.04	-0.03 (0.06)	6.05E-01	1085	0.04	0.02 (0.03)	6.21E-01	1002	8.35E-01	7.74E-01	4.94E-01
rs2271293	A	EA	0.12	0.00 (0.01)	8.03E-01	7940	0.12	-0.01 (0.01)	6.87E-02	13884	9.60E-02	1.85E-01	4.37E-01
LCAT		AA	0.07	0.01 (0.02)	6.24E-01	3128	0.07	0.01 (0.01)	6.20E-01	5766	5.03E-01	7.84E-01	8.47E-01
		AI	0.25	-0.05 (0.02)	5.79E-03	2608	0.24	0.01 (0.01)	6.50E-01	4217	2.08E-01	2.00E-02	1.25E-02
		MA/H	0.12	0.02 (0.04)	5.07E-01	1082	0.15	0.01 (0.02)	6.56E-01	1770	5.10E-01	7.27E-01	6.52E-01
rs2228671	T	EA	0.12	0.01 (0.02)	7.49E-01	2692	0.12	0.00 (0.01)	8.92E-01	3509	9.69E-01	9.42E-01	7.30E-01
LDLR		AA	0.04	0.01 (0.05)	8.13E-01	1235	0.04	-0.04 (0.03)	1.51E-01	1312	2.38E-01	3.46E-01	3.93E-01
		AI	--	--	--	--	--	--	--	--	--	--	--
		MA/H	0.08	0.07 (0.04)	9.58E-02	1120	0.08	0.02 (0.02)	4.37E-01	1051	1.53E-01	1.85E-01	2.48E-01
rs6511720	T	EA	0.12	0.01 (0.01)	5.25E-01	7707	0.12	-0.01 (0.01)	1.76E-01	13393	4.97E-01	3.27E-01	1.83E-01
LDLR		AA	0.14	-0.02 (0.02)	3.59E-01	2817	0.13	-0.01 (0.01)	3.17E-01	5420	1.81E-01	3.98E-01	8.20E-01
		AI	0.05	0.11 (0.04)	2.80E-03	2605	0.04	-0.03 (0.03)	1.83E-01	4216	5.54E-01	4.70E-03	1.29E-03
		MA/H	0.10	0.22 (0.08)	6.69E-03	182	0.09	-0.10 (0.04)	6.50E-03	852	1.62E-01	6.21E-04	3.46E-04
rs261332	A	EA	0.20	0.00 (0.01)	9.40E-01	4439	0.20	0.02 (0.01)	5.08E-02	4926	1.32E-01	1.48E-01	2.13E-01
LIPC		AA	0.24	0.00 (0.02)	8.26E-01	1399	0.25	0.03 (0.02)	8.88E-02	1827	1.40E-01	2.29E-01	3.83E-01
		AI	--	--	--	--	--	--	--	--	--	--	--
		MA/H	0.20	-0.06 (0.06)	3.83E-01	179	0.16	-0.10 (0.10)	3.27E-01	80	2.05E-01	4.23E-01	7.34E-01
rs4775041	C	EA	0.29	0.01 (0.01)	1.15E-01	8391	0.29	0.01 (0.01)	1.96E-01	9843	4.92E-02	1.25E-01	5.91E-01
LIPC		AA	0.14	0.05 (0.02)	1.22E-02	3211	0.13	0.02 (0.01)	1.09E-01	4353	7.54E-03	1.20E-02	1.91E-01
		AI	0.21	0.03 (0.02)	1.37E-01	2518	0.21	0.02 (0.01)	2.10E-01	4088	5.77E-02	1.51E-01	6.69E-01

		MA/H	0.18	0.00 (0.03)	9.44E-01	1139	0.18	0.02 (0.01)	3.07E-01	1052	3.77E-01	5.93E-01	6.06E-01
rs2156552	T	EA	0.17	0.00 (0.01)	8.33E-01	8420	0.16	0.00 (0.01)	7.60E-01	9873	7.11E-01	9.34E-01	9.95E-01
LIPG		AA	0.05	0.05 (0.03)	8.41E-02	3210	0.04	-0.02 (0.02)	2.40E-01	4362	9.41E-01	1.13E-01	3.69E-02
		AI	0.04	0.06 (0.04)	9.81E-02	2613	0.04	-0.05 (0.03)	1.58E-01	4083	9.98E-01	9.39E-02	2.96E-02
		MA/H	0.07	-0.02 (0.04)	6.00E-01	1131	0.08	-0.01 (0.02)	7.02E-01	1047	5.73E-01	8.10E-01	7.48E-01
rs2197089	T	EA	0.55	-0.04 (0.01)	4.01E-06	8424	0.56	-0.03 (0.01)	6.43E-09	14301	1.51E-13	1.12E-12	5.24E-01
LPL		AA	0.78	-0.01 (0.01)	6.75E-01	3223	0.78	-0.02 (0.01)	3.44E-02	5893	4.12E-02	9.77E-02	4.87E-01
		AI	0.41	-0.05 (0.02)	1.48E-03	2615	0.41	-0.05 (0.01)	7.72E-06	4217	4.10E-08	2.84E-07	9.59E-01
		MA/H	0.46	-0.06 (0.02)	1.05E-02	1141	0.48	-0.02 (0.01)	4.57E-02	1824	4.21E-03	5.12E-03	1.25E-01
rs328	C	EA	0.90	0.11 (0.01)	6.75E-16	8241	0.90	0.07 (0.01)	7.23E-15	14057	7.80E-28	4.52E-28	1.29E-02
LPL		AA	0.93	0.11 (0.03)	1.56E-04	2658	0.93	0.06 (0.01)	5.75E-05	5097	1.16E-07	2.37E-07	1.22E-01
		AI	0.97	0.04 (0.05)	4.11E-01	2618	0.97	0.09 (0.04)	1.74E-02	4225	1.82E-02	4.21E-02	3.84E-01
		MA/H	0.94	0.10 (0.05)	3.55E-02	1131	0.93	0.05 (0.02)	2.58E-02	1818	4.08E-03	9.12E-03	2.85E-01
rs6586891	A	EA	0.65	-0.05 (0.01)	1.04E-07	7008	0.65	-0.03 (0.01)	4.84E-06	12569	6.41E-12	2.01E-11	1.58E-01
LPL		AA	0.84	-0.01 (0.02)	6.03E-01	2525	0.84	-0.03 (0.01)	5.29E-03	5007	8.51E-03	1.78E-02	2.89E-01
		AI	0.45	-0.02 (0.02)	1.67E-01	2605	0.45	-0.02 (0.01)	1.29E-01	4210	4.17E-02	1.22E-01	8.00E-01
		MA/H	0.55	-0.01 (0.05)	8.79E-01	183	0.58	-0.03 (0.02)	1.63E-01	852	1.84E-01	3.74E-01	6.54E-01
rs6102059	T	EA	0.30	-0.01 (0.01)	5.00E-01	4442	0.30	-0.01 (0.01)	4.34E-01	4951	3.01E-01	5.86E-01	9.95E-01
MAFB		AA	0.41	-0.02 (0.02)	3.69E-01	1412	0.42	0.02 (0.01)	1.39E-01	1832	5.27E-01	2.24E-01	1.08E-01
		AI	--	--	--	--	--	--	--	--	--	--	--
		MA/H	0.26	0.06 (0.06)	3.42E-01	182	0.30	0.00 (0.08)	9.68E-01	80	4.38E-01	6.36E-01	5.81E-01
rs17145738	T	EA	0.12	-0.07 (0.01)	9.92E-10	8433	0.12	-0.06 (0.01)	2.48E-15	14308	1.98E-23	1.81E-22	4.72E-01
MLXIPL		AA	0.09	-0.02 (0.02)	2.95E-01	3224	0.09	-0.02 (0.01)	5.35E-02	5884	2.81E-02	8.96E-02	9.89E-01
		AI	0.07	-0.07 (0.03)	1.52E-02	2615	0.07	-0.05 (0.02)	4.17E-02	4211	2.04E-03	6.59E-03	4.70E-01
		MA/H	0.08	-0.04 (0.04)	3.84E-01	1142	0.07	-0.07 (0.02)	9.54E-04	1828	7.74E-04	2.91E-03	5.45E-01
rs2338104	C	EA	0.46	0.01 (0.01)	2.74E-01	8422	0.46	-0.01 (0.01)	2.23E-01	9877	7.33E-01	2.62E-01	1.09E-01
MMAB-		AA	0.28	0.00 (0.01)	9.32E-01	3211	0.26	-0.01 (0.01)	5.07E-01	4366	6.07E-01	8.00E-01	6.70E-01
MVK		AI	0.57	0.00 (0.02)	8.40E-01	2598	0.58	-0.02 (0.01)	7.57E-02	4047	1.93E-01	2.02E-01	2.21E-01
		MA/H	0.53	0.01 (0.02)	8.02E-01	1137	0.52	0.02 (0.01)	1.55E-01	1055	1.69E-01	3.52E-01	6.56E-01
rs11206510	T	EA	0.81	0.00 (0.01)	9.15E-01	8433	0.81	0.01 (0.01)	1.17E-01	14307	1.69E-01	2.90E-01	4.46E-01
PCSK9		AA	0.86	0.03 (0.02)	5.49E-02	3217	0.86	0.00 (0.01)	6.56E-01	5893	1.78E-01	1.43E-01	1.50E-01
		AI	0.94	0.01 (0.03)	7.26E-01	2618	0.94	0.10 (0.02)	9.22E-05	4221	7.63E-04	4.46E-04	4.31E-02
		MA/H	0.90	-0.01 (0.04)	8.73E-01	1142	0.87	-0.01 (0.02)	6.00E-01	1827	5.85E-01	8.61E-01	9.65E-01
rs11591147	T	EA	0.02	0.00 (0.03)	9.80E-01	8909	0.02	-0.01 (0.02)	6.62E-01	14936	7.11E-01	9.09E-01	8.16E-01
PCSK9		AA	<0.01	-0.13 (0.12)	3.14E-01	2842	<0.01	-0.09 (0.05)	4.92E-02	5342	2.81E-02	8.70E-02	8.01E-01
		AI	0.01	0.21 (0.14)	1.20E-01	1069	0.01	-0.13 (0.08)	1.06E-01	2344	5.32E-01	8.10E-02	3.14E-02

		MA/H	<0.01	-0.22 (0.23)	3.46E-01	953	0.01	0.03 (0.07)	6.50E-01	1736	8.72E-01	5.78E-01	3.01E-01
rs7679	T	EA	0.82	-0.01 (0.01)	3.04E-01	5445	0.82	-0.01 (0.01)	4.05E-01	6092	1.93E-01	4.17E-01	8.17E-01
PLTP		AA	0.96	0.02 (0.03)	4.75E-01	2534	0.96	-0.04 (0.03)	1.24E-01	3481	4.45E-01	2.37E-01	1.30E-01
		AI	0.95	-0.01 (0.03)	7.27E-01	2617	0.95	0.01 (0.03)	6.18E-01	4082	8.51E-01	8.31E-01	5.63E-01
		MA/H	0.88	0.04 (0.08)	6.13E-01	184	0.92	-0.11 (0.13)	3.80E-01	83	9.47E-01	5.98E-01	3.12E-01
rs1501908	G	EA	0.63	0.01 (0.01)	5.68E-01	6868	0.64	0.00 (0.01)	6.93E-01	7823	5.17E-01	7.86E-01	8.04E-01
TIMD4		AA	0.37	0.01 (0.01)	2.95E-01	3219	0.37	0.00 (0.01)	9.92E-01	4366	5.96E-01	5.77E-01	3.66E-01
		AI	0.87	0.03 (0.02)	1.38E-01	2601	0.88	0.03 (0.02)	8.66E-02	4055	2.38E-02	7.68E-02	8.90E-01
		MA/H	0.77	0.01 (0.03)	8.50E-01	1140	0.76	-0.01 (0.01)	2.75E-01	1060	3.64E-01	5.42E-01	5.26E-01
rs2075650	A	EA	0.87	0.00 (0.03)	9.37E-01	1283	0.88	0.00 (0.03)	8.88E-01	1797	8.74E-01	9.87E-01	9.73E-01
TOMM40		AA	0.87	-0.06 (0.04)	1.55E-01	572	0.86	-0.02 (0.04)	6.70E-01	433	1.98E-01	3.32E-01	4.60E-01
		AI	--	--	--	--	--	--	--	--	--	--	--
		MA/H	0.90	0.04 (0.08)	6.01E-01	185	0.92	-0.10 (0.12)	4.37E-01	83	9.97E-01	6.45E-01	3.49E-01
rs2954029	A	EA	0.54	0.06 (0.01)	9.84E-10	5877	0.53	0.03 (0.01)	1.27E-06	6803	1.26E-13	5.92E-14	1.50E-02
TRIB1		AA	0.67	-0.07 (0.03)	1.42E-02	686	0.68	0.02 (0.01)	1.80E-01	890	7.71E-01	2.01E-02	5.46E-03
		AI	--	--	--	--	--	--	--	--	--	--	--
		MA/H	0.61	0.05 (0.03)	7.34E-02	959	0.63	0.03 (0.01)	3.62E-03	978	7.13E-04	2.91E-03	6.46E-01
rs471364	A	EA	0.89	-0.01 (0.01)	3.82E-01	7940	0.88	0.01 (0.01)	3.72E-01	9470	8.10E-01	4.58E-01	2.20E-01
TTC39B		AA	0.81	0.03 (0.02)	8.87E-02	3129	0.81	-0.02 (0.01)	8.35E-02	4242	5.66E-01	5.25E-02	1.83E-02
		AI	0.97	0.00 (0.04)	9.45E-01	2602	0.97	0.04 (0.03)	2.18E-01	4067	3.53E-01	4.67E-01	4.18E-01
		MA/H	0.92	0.03 (0.04)	4.77E-01	1087	0.92	0.04 (0.02)	7.06E-02	1001	5.28E-02	1.51E-01	8.74E-01

*Abbreviations: RE=race/ethnicity: EA=European American; AA=African American; AI=American Indian; MA/H=Mexican American/Hispanic; CAF = coding allele frequency; SE=standard error.

†P-value for SNP-phenotype association in the sex-combined meta-analysis, not allowing for different effects by sex.

‡P-value for SNP-phenotype association in the sex-combined meta-analysis, allowing for different effects by sex.

§P-value for heterogeneity by sex.

||Data not available.

Supplementary Table 3. Sex-specific associations of GWAS-identified lipid SNPs with LDL-C levels in the PAGE study.

			Males				Females						
SNP/Gene	Coded Allele	Population*	CAF	β (SE)	P	N	CAF	β (SE)	P	N	P_{comb}†	P_{diff}‡	P_{het}§
rs1883025 *ABCA1*	A	EA	0.25	-1.94 (0.76)	1.10E-02	5571	0.25	-1.28 (0.59)	3.02E-02	6521	1.08E-03	3.77E-03	4.96E-01
		AA	0.34	-4.23 (2.45)	8.41E-02	525	0.34	-0.43 (1.06)	6.83E-01	694	2.89E-01	2.07E-01	1.54E-01
		AI	--‖	--	--	--	--	--	--	--	--	--	--
		MA/H	0.28	0.99 (1.89)	6.02E-01	747	0.26	0.15 (0.92)	8.74E-01	794	7.11E-01	8.62E-01	6.89E-01
rs3890182 *ABCA1*	A	EA	0.12	-1.04 (0.83)	2.06E-01	8092	0.12	-1.26 (0.62)	4.08E-02	12430	1.66E-02	5.55E-02	8.34E-01
		AA	0.12	-0.92 (1.43)	5.21E-01	3044	0.12	-0.11 (0.95)	9.09E-01	5552	6.52E-01	8.09E-01	6.38E-01
		AI	0.04	3.91 (2.47)	1.13E-01	2615	0.04	1.79 (1.86)	3.36E-01	4210	8.54E-02	1.80E-01	4.92E-01
		MA/H	0.08	2.53 (3.01)	4.02E-01	919	0.09	-0.71 (1.36)	6.02E-01	1591	8.96E-01	6.14E-01	3.28E-01
rs4149268 *ABCA1*	A	EA	0.37	-0.81 (0.68)	2.29E-01	5573	0.37	-0.09 (0.53)	8.65E-01	6393	3.83E-01	4.78E-01	3.98E-01
		AA	0.67	0.22 (1.38)	8.74E-01	1925	0.68	-0.51 (0.87)	5.60E-01	2520	6.83E-01	8.33E-01	6.56E-01
		AI	--	--	--	--	--	--	--	--	--	--	--
		MA/H	0.31	1.70 (1.72)	3.22E-01	886	0.32	-0.11 (0.85)	8.95E-01	825	7.48E-01	6.07E-01	3.44E-01
rs6544713 *ABCG8*	T	EA	0.31	4.31 (0.68)	2.18E-10	6024	0.31	2.32 (0.61)	1.55E-04	9672	1.64E-12	1.33E-12	2.94E-02
		AA	0.17	2.75 (1.31)	3.67E-02	2809	0.17	1.47 (0.98)	1.36E-01	5296	1.45E-02	3.72E-02	4.36E-01
		AI	0.09	4.82 (1.64)	3.33E-03	2611	0.09	4.90 (1.28)	1.30E-04	4204	1.43E-06	8.81E-06	9.68E-01
		MA/H	0.16	2.76 (4.78)	5.64E-01	181	0.22	0.65 (2.21)	7.70E-01	808	6.12E-01	8.11E-01	6.88E-01
rs1748195 *ANGPLT3*	C	EA	0.67	1.94 (0.72)	6.69E-03	5405	0.66	1.57 (0.50)	1.72E-03	9146	3.76E-05	1.85E-04	6.69E-01
		AA	0.34	2.24 (1.50)	1.36E-01	1568	0.35	1.17 (0.78)	1.34E-01	3826	4.38E-02	1.07E-01	5.27E-01
		AI	--	--	--	--	0.61	-0.37 (6.47)	9.55E-01	125	9.55E-01	--	--
		MA/H	0.60	1.92 (1.76)	2.76E-01	748	0.59	0.84 (0.73)	2.46E-01	1523	1.37E-01	2.82E-01	5.74E-01
rs2967605 *ANGPLT4*	A	EA	0.18	-0.08 (0.68)	9.03E-01	8093	0.18	1.04 (0.55)	5.89E-02	9545	1.66E-01	1.67E-01	1.97E-01
		AA	0.21	-0.11 (1.15)	9.24E-01	3043	0.21	-0.09 (0.82)	9.12E-01	4160	8.84E-01	9.89E-01	9.89E-01
		AI	0.30	-0.61 (0.97)	5.28E-01	2611	0.29	-1.37 (0.74)	6.56E-02	4075	6.48E-02	1.50E-01	5.38E-01
		MA/H	0.23	0.24 (1.89)	8.97E-01	926	0.24	1.46 (0.89)	1.01E-01	867	1.24E-01	2.59E-01	5.60E-01
rs28927680 *APOA1/C3/ A4/A5*	G	EA	0.93	1.00 (1.03)	3.31E-01	8795	0.93	-0.41 (0.79)	6.06E-01	13251	8.53E-01	5.46E-01	2.78E-01
		AA	0.84	1.01 (1.26)	4.24E-01	3330	0.84	0.33 (0.83)	6.92E-01	6007	4.40E-01	6.71E-01	6.54E-01
		AI	0.87	-3.11 (1.31)	1.75E-02	2614	0.87	-1.51 (0.93)	1.02E-01	4211	6.78E-03	1.56E-02	3.20E-01
		MA/H	0.85	1.90 (2.33)	4.15E-01	928	0.87	-0.37 (1.06)	7.25E-01	1603	9.87E-01	6.74E-01	3.75E-01

rs3135506 *APOA5*	C	EA	0.06	-1.32 (1.13)	2.41E-01	7832	0.06	1.15 (0.93)	2.17E-01	9257	8.38E-01	2.35E-01	9.10E-02
		AA	0.06	-0.79 (2.35)	7.37E-01	2500	0.06	1.98 (1.55)	2.03E-01	3421	3.81E-01	4.20E-01	3.26E-01
		AI	0.12	2.96 (1.32)	2.44E-02	2623	0.13	1.35 (0.94)	1.51E-01	4088	1.33E-02	2.83E-02	3.19E-01
		MA/H	0.14	2.11 (2.42)	3.83E-01	920	0.13	0.64 (1.16)	5.79E-01	861	3.80E-01	5.85E-01	5.84E-01
rs964184 *APOA1/C3/ A4/A5*	C	EA	0.86	-0.95 (1.14)	4.05E-01	4086	0.86	-2.94 (1.02)	4.18E-03	7516	7.21E-03	1.17E-02	1.95E-01
		AA	0.80	0.40 (2.25)	8.57E-01	1023	0.80	-2.03 (1.41)	1.50E-01	3098	2.60E-01	3.48E-01	3.58E-01
		AI	--	--	--	--	0.78	-0.92 (5.71)	8.72E-01	125	8.72E-01	--	--
		MA/H	--	--	--	--	0.75	-1.00 (2.20)	6.50E-01	731	6.50E-01	--	--
rs562338 *APOB*	T	EA	0.19	-5.32 (0.73)	3.30E-13	6858	0.19	-5.96 (0.66)	1.62E-19	10930	4.73E-31	5.16E-30	5.14E-01
		AA	0.59	-3.08 (1.04)	2.93E-03	2514	0.59	-2.15 (0.80)	7.17E-03	4852	7.95E-05	3.21E-04	4.78E-01
		AI	0.08	-3.93 (1.62)	1.53E-02	2618	0.08	-5.35 (1.22)	1.08E-05	4207	6.56E-07	3.26E-06	4.83E-01
		MA/H	0.16	-6.58 (5.03)	1.91E-01	181	0.19	-1.50 (2.34)	5.21E-01	810	2.57E-01	3.46E-01	3.59E-01
rs693 *APOB*	T	EA	0.49	2.77 (0.59)	2.43E-06	6425	0.50	3.83 (0.52)	2.68E-13	10544	8.53E-18	3.53E-17	1.76E-01
		AA	0.24	2.80 (1.25)	2.57E-02	2451	0.23	0.33 (0.93)	7.20E-01	4744	1.07E-01	7.78E-02	1.14E-01
		AI	0.33	4.50 (0.93)	1.32E-06	2616	0.33	3.53 (0.68)	1.95E-07	4198	1.73E-12	1.05E-11	3.99E-01
		MA/H	0.37	4.22 (3.92)	2.82E-01	181	0.41	1.95 (1.97)	3.22E-01	809	1.71E-01	3.43E-01	6.05E-01
rs754523 *APOB*	T	EA	0.68	-3.04 (0.63)	1.43E-06	6428	0.68	-4.00 (0.61)	7.10E-11	7686	9.38E-16	5.07E-15	2.77E-01
		AA	0.78	-2.09 (1.26)	9.59E-02	2445	0.79	-2.17 (1.10)	4.90E-02	3342	9.97E-03	3.60E-02	9.61E-01
		AI	0.69	-3.85 (0.96)	6.21E-05	2614	0.68	-3.99 (0.70)	1.28E-08	4073	3.53E-12	3.00E-11	9.05E-01
		MA/H	0.71	-11.68 (4.02)	3.66E-03	179	0.69	2.75 (6.67)	6.80E-01	78	2.28E-02	1.34E-02	6.38E-02
rs10402271 *APOE/C1/ C4*	T	EA	0.67	-2.29 (0.69)	8.74E-04	5430	0.67	-2.23 (0.69)	1.27E-03	6548	3.63E-06	2.16E-05	9.51E-01
		AA	0.84	1.33 (1.94)	4.93E-01	1609	0.84	-3.09 (1.69)	6.77E-02	2165	3.55E-01	1.49E-01	8.58E-02
		AI	--	--	--	--	--	--	--	--	--	--	--
		MA/H	0.61	1.06 (4.12)	7.97E-01	182	0.63	5.08 (5.96)	3.94E-01	79	4.86E-01	6.73E-01	5.79E-01
rs4420638 *APOE/C1/ C4*	A	EA	0.82	-4.62 (0.77)	1.65E-09	6925	0.82	-4.79 (0.56)	9.07E-18	11179	9.57E-26	1.16E-24	8.60E-01
		AA	0.80	1.59 (1.54)	3.04E-01	1837	0.80	-0.01 (0.90)	9.92E-01	4307	6.11E-01	5.90E-01	3.72E-01
		AI	0.92	-0.81 (1.66)	6.25E-01	2621	0.92	-5.51 (1.23)	7.95E-06	4205	1.06E-04	4.08E-05	2.30E-02
		MA/H	0.90	-5.64 (2.99)	5.94E-02	708	0.89	-2.60 (1.22)	3.35E-02	1476	7.33E-03	1.76E-02	3.48E-01
rs4803750 *BCL3*	A	EA	0.93	6.01 (1.34)	7.16E-06	5451	0.93	5.19 (1.25)	3.53E-05	6566	1.15E-09	7.92E-09	6.53E-01
		AA	0.92	6.97 (2.71)	1.01E-02	1613	0.92	-1.97 (2.29)	3.90E-01	2182	3.15E-01	2.53E-02	1.18E-02
		AI	--	--	--	--	--	--	--	--	--	--	--
		MA/H	0.86	6.06 (5.57)	2.77E-01	182	0.84	7.21 (8.75)	4.10E-01	79	1.74E-01	3.94E-01	9.11E-01
rs646776 *CELSR2/ PSCR1/ SORT1*	A	EA	0.78	5.79 (0.70)	8.93E-17	6590	0.79	6.59 (0.67)	1.03E-22	7893	1.02E-37	1.07E-36	4.11E-01
		AA	0.64	3.27 (1.05)	1.88E-03	2739	0.65	5.07 (0.90)	1.96E-08	3786	3.28E-10	1.10E-09	1.93E-01
		AI	0.80	4.55 (1.62)	4.87E-03	1074	0.80	6.93 (1.31)	1.18E-07	1741	3.93E-09	1.51E-08	2.52E-01
		MA/H	0.80	10.18 (4.91)	3.83E-02	181	0.74	0.37 (7.15)	9.59E-01	78	8.25E-02	1.17E-01	2.58E-01

rs599839	A	EA	0.77	5.65 (0.68)	6.79E-17	6866	0.77	6.06 (0.66)	4.58E-20	8062	2.63E-35	3.43E-34	6.64E-01
CELSR2/		AA	0.28	0.81 (1.13)	4.70E-01	2514	0.28	1.95 (1.01)	5.27E-02	3459	5.43E-02	1.18E-01	4.50E-01
PSCR1/		AI	0.78	5.93 (1.03)	1.03E-08	2595	0.78	6.43 (0.79)	3.15E-16	4066	2.11E-23	2.31E-22	6.96E-01
SORT1		MA/H	0.77	8.35 (4.66)	7.31E-02	177	0.72	1.39 (6.81)	8.38E-01	79	1.11E-01	1.96E-01	3.99E-01
rs12596776	C	EA	0.90	1.40 (1.14)	2.17E-01	5557	0.90	-0.27 (0.86)	7.56E-01	6391	6.19E-01	4.45E-01	2.42E-01
CETP		AA	0.94	0.56 (2.76)	8.41E-01	1928	0.95	3.12 (1.71)	6.75E-02	2512	9.67E-02	1.84E-01	4.29E-01
		AI	--	--	--	--	--	--	--	--	--	--	--
		MA/H	0.94	-2.73 (3.15)	3.85E-01	920	0.94	0.36 (1.63)	8.27E-01	871	8.36E-01	6.69E-01	3.83E-01
rs1566439	A	EA	0.60	0.64 (0.61)	2.88E-01	6670	0.60	0.88 (0.46)	5.71E-02	8075	3.10E-02	9.32E-02	7.59E-01
CETP		AA	0.78	-0.43 (1.46)	7.68E-01	2136	0.78	0.80 (0.96)	4.03E-01	2874	5.91E-01	6.75E-01	4.81E-01
		AI	--	--	--	--	--	--	--	--	--	--	--
		MA/H	0.54	-1.73 (1.58)	2.72E-01	923	0.54	-0.88 (0.78)	2.58E-01	872	1.34E-01	2.89E-01	6.26E-01
rs1864163	A	EA	0.24	-1.74 (3.76)	6.43E-01	181	0.21	-1.70 (5.55)	7.60E-01	109	5.79E-01	8.57E-01	9.95E-01
CETP		AA	0.27	-1.30 (3.62)	7.18E-01	367	0.24	5.30 (6.95)	4.46E-01	84	9.74E-01	7.01E-01	3.99E-01
		AI	--	--	--	--	--	--	--	--	--	--	--
		MA/H	0.27	1.87 (4.18)	6.54E-01	182	0.30	-5.05 (7.14)	4.79E-01	80	9.76E-01	7.04E-01	4.03E-01
rs3764261	T	EA	0.32	-0.86 (0.56)	1.22E-01	8265	0.33	-0.85 (0.45)	5.57E-02	9916	1.39E-02	4.84E-02	9.90E-01
CETP		AA	0.31	-1.06 (0.99)	2.82E-01	3230	0.32	-1.21 (0.70)	8.26E-02	4434	4.15E-02	1.24E-01	9.03E-01
		AI	0.31	0.09 (0.95)	9.23E-01	2599	0.31	-0.48 (0.71)	4.98E-01	4059	6.28E-01	7.91E-01	6.29E-01
		MA/H	0.34	-0.85 (1.78)	6.35E-01	887	0.32	0.41 (0.84)	6.26E-01	825	8.13E-01	7.93E-01	5.23E-01
rs9989419	A	EA	0.40	0.38 (0.56)	4.92E-01	7568	0.40	0.33 (0.54)	5.45E-01	8856	3.61E-01	6.58E-01	9.43E-01
CETP		AA	0.59	-0.37 (1.00)	7.08E-01	2797	0.60	-0.03 (0.87)	9.68E-01	3889	7.83E-01	9.32E-01	7.97E-01
		AI	0.26	1.63 (1.01)	1.06E-01	2611	0.25	1.92 (0.77)	1.23E-02	4072	2.99E-03	1.19E-02	8.23E-01
		MA/H	0.34	6.00 (3.86)	1.20E-01	180	0.40	-2.19 (6.53)	7.37E-01	79	2.43E-01	2.82E-01	2.80E-01
rs16996148	T	EA	0.08	-3.44 (0.96)	3.50E-04	8047	0.08	-2.11 (0.76)	5.63E-03	12374	1.14E-05	3.60E-05	2.79E-01
CILP2/		AA	0.15	0.82 (1.29)	5.26E-01	3015	0.15	0.49 (0.85)	5.68E-01	5528	4.09E-01	6.95E-01	8.31E-01
PBX4/		AI	0.04	-3.94 (2.31)	8.82E-02	2620	0.04	1.24 (1.76)	4.80E-01	4210	6.38E-01	1.82E-01	7.43E-02
NCAN1		MA/H	0.06	-2.73 (3.53)	4.40E-01	927	0.06	-1.69 (1.56)	2.79E-01	1605	1.93E-01	4.13E-01	7.87E-01
rs174547	T	EA	0.66	2.01 (0.64)	1.64E-03	6504	0.66	1.45 (0.49)	3.26E-03	7464	2.13E-05	9.22E-05	4.82E-01
FADS1		AA	0.91	-0.26 (1.60)	8.74E-01	3014	0.92	0.09 (1.17)	9.39E-01	4119	9.75E-01	9.84E-01	8.62E-01
		AI	0.17	7.41 (1.30)	1.15E-08	2214	0.15	6.15 (1.03)	2.12E-09	3519	1.77E-16	1.31E-15	4.45E-01
		MA/H	0.39	1.53 (1.65)	3.52E-01	883	0.40	1.36 (0.77)	7.79E-02	828	4.65E-02	1.37E-01	9.24E-01
rs2144300	T	EA	0.60	-0.17 (0.54)	7.58E-01	7999	0.60	-0.09 (0.42)	8.37E-01	12263	7.25E-01	9.34E-01	9.06E-01
GALNT2		AA	0.14	-0.74 (1.33)	5.80E-01	3026	0.16	0.51 (0.81)	5.32E-01	5551	8.06E-01	7.06E-01	4.25E-01
		AI	0.55	0.93 (0.87)	2.86E-01	2609	0.55	0.86 (0.65)	1.86E-01	4196	8.97E-02	2.36E-01	9.50E-01
		MA/H	0.57	-0.11 (1.63)	9.45E-01	930	0.55	-1.04 (0.73)	1.54E-01	1604	1.84E-01	3.61E-01	6.02E-01

rs1260326	T	EA	0.42	0.95 (0.61)	1.20E-01	6645	0.41	0.09 (0.44)	8.40E-01	11028	2.81E-01	2.92E-01	2.54E-01
GCKR		AA	0.17	-2.16 (2.33)	3.53E-01	723	0.15	1.28 (1.13)	2.59E-01	2429	5.42E-01	3.43E-01	1.84E-01
		AI	--	--	--	--	0.28	7.25 (6.26)	2.47E-01	124	2.47E-01	--	--
		MA/H	0.32	-2.11 (1.86)	2.59E-01	738	0.33	0.30 (0.78)	7.03E-01	1514	9.35E-01	4.91E-01	2.34E-01
rs780094	A	EA	0.40	0.65 (0.60)	2.80E-01	6430	0.40	0.78 (0.58)	1.83E-01	7695	8.77E-02	2.30E-01	8.76E-01
GCKR		AA	0.19	-0.08 (1.32)	9.49E-01	2449	0.18	1.90 (1.19)	1.11E-01	3348	2.53E-01	2.80E-01	2.65E-01
		AI	0.21	-0.99 (1.13)	3.83E-01	2617	0.20	1.61 (0.86)	6.05E-02	4084	3.31E-01	1.17E-01	6.76E-02
		MA/H	0.34	-0.70 (4.31)	8.72E-01	181	0.33	-1.65 (6.95)	8.12E-01	79	7.93E-01	9.59E-01	9.07E-01
rs12654264	A	EA	0.62	-4.03 (0.54)	8.25E-14	8088	0.62	-1.17 (0.42)	5.98E-03	12424	1.23E-11	1.72E-14	3.06E-05
HMGCR		AA	0.67	-0.79 (0.97)	4.15E-01	3040	0.67	-2.61 (0.67)	8.80E-05	5544	2.18E-04	3.26E-04	1.23E-01
		AI	0.57	-1.93 (0.93)	3.71E-02	2612	0.59	-0.95 (0.68)	1.62E-01	4210	1.82E-02	4.28E-02	3.94E-01
		MA/H	0.61	-4.05 (1.75)	2.05E-02	919	0.62	-1.06 (0.76)	1.66E-01	1589	2.80E-02	2.61E-02	1.17E-01
rs2650000	T	EA	0.35	1.01 (0.56)	7.30E-02	7615	0.35	1.08 (0.45)	1.72E-02	9149	2.88E-03	1.17E-02	9.19E-01
HNF1A		AA	0.12	0.93 (1.46)	5.23E-01	2961	0.12	-0.13 (1.03)	9.03E-01	4042	7.88E-01	8.09E-01	5.54E-01
		AI	0.41	1.35 (0.89)	1.31E-01	2613	0.41	0.50 (0.66)	4.52E-01	4085	1.33E-01	2.41E-01	4.44E-01
		MA/H	0.36	0.66 (1.71)	7.01E-01	924	0.36	2.36 (0.82)	4.20E-03	866	6.04E-03	1.54E-02	3.70E-01
rs1800961	T	EA	0.03	-1.34 (1.62)	4.05E-01	7611	0.03	-4.08 (1.23)	8.77E-04	9160	1.61E-03	2.78E-03	1.78E-01
HNF4A		AA	0.01	-10.70 (5.75)	6.26E-02	2957	0.01	-8.22 (4.24)	5.25E-02	4038	7.69E-03	2.69E-02	7.29E-01
		AI	0.03	1.00 (2.39)	6.76E-01	2619	0.03	-2.55 (1.92)	1.83E-01	4087	4.37E-01	3.78E-01	2.47E-01
		MA/H	0.04	2.66 (4.15)	5.22E-01	887	0.04	-1.09 (2.00)	5.86E-01	826	8.32E-01	7.03E-01	4.16E-01
rs2271293	A	EA	0.12	-1.33 (0.83)	1.10E-01	7632	0.12	-0.84 (0.64)	1.87E-01	12042	4.36E-02	1.17E-01	6.36E-01
LCAT		AA	0.07	-2.45 (1.86)	1.86E-01	2958	0.07	-1.19 (1.15)	3.02E-01	5437	1.15E-01	2.45E-01	5.61E-01
		AI	0.25	-1.89 (1.02)	6.39E-02	2610	0.24	0.29 (0.77)	7.06E-01	4208	4.17E-01	1.67E-01	8.77E-02
		MA/H	0.12	-3.86 (2.52)	1.26E-01	888	0.15	-0.44 (1.04)	6.74E-01	1554	3.31E-01	2.84E-01	2.10E-01
rs2228671	T	EA	0.12	-1.50 (1.45)	3.01E-01	2476	0.12	0.54 (0.89)	5.40E-01	3254	9.86E-01	4.85E-01	2.29E-01
LDLR		AA	0.04	4.08 (4.48)	3.63E-01	1080	0.04	4.67 (2.33)	4.45E-02	1122	2.76E-02	8.78E-02	9.06E-01
		AI	--	--	--	--	--	--	--	--	--	--	--
		MA/H	0.08	5.46 (2.91)	6.08E-02	917	0.08	0.36 (1.44)	8.00E-01	865	2.91E-01	1.67E-01	1.16E-01
rs6511720	T	EA	0.12	-7.31 (0.83)	1.06E-18	7566	0.12	-7.58 (0.76)	1.17E-23	11736	1.04E-40	1.55E-39	8.14E-01
LDLR		AA	0.14	-7.50 (1.42)	1.29E-07	2803	0.13	-8.00 (1.08)	1.12E-13	5284	8.36E-20	8.76E-19	7.80E-01
		AI	0.05	-5.46 (2.20)	1.29E-02	2607	0.04	-0.22 (1.56)	8.86E-01	4207	1.19E-01	4.50E-02	5.20E-02
		MA/H	0.10	-4.51 (5.92)	4.46E-01	181	0.09	-6.64 (2.97)	2.54E-02	809	1.93E-02	6.15E-02	7.48E-01
rs261332	A	EA	0.20	0.18 (0.93)	8.46E-01	4340	0.20	-1.84 (0.97)	5.91E-02	4876	2.45E-01	1.65E-01	1.34E-01
LIPC		AA	0.24	-1.85 (1.80)	3.02E-01	1389	0.25	-1.68 (1.66)	3.10E-01	1816	1.48E-01	3.51E-01	9.45E-01
		AI	--	--	--	--	--	--	--	--	--	--	--
		MA/H	0.20	6.12 (4.66)	1.88E-01	178	0.16	13.40 (8.22)	1.03E-01	78	5.15E-02	1.12E-01	4.41E-01

rs4775041 *LIPC*	C	EA	0.29	0.97 (0.58)	9.49E-02	8066	0.29	-0.14 (0.47)	7.57E-01	9524	4.21E-01	2.36E-01	1.35E-01
		AA	0.14	-2.06 (1.37)	1.33E-01	3036	0.13	0.22 (0.97)	8.24E-01	4142	4.94E-01	3.16E-01	1.75E-01
		AI	0.21	-0.23 (1.04)	8.26E-01	2620	0.21	-1.64 (0.82)	4.63E-02	4086	8.89E-02	1.34E-01	2.88E-01
		MA/H	0.18	1.41 (2.07)	4.98E-01	927	0.18	-1.31 (1.00)	1.91E-01	866	3.78E-01	3.38E-01	2.38E-01
rs2156552 *LIPG*	T	EA	0.17	0.63 (0.70)	3.72E-01	8093	0.16	0.95 (0.57)	9.72E-02	9553	6.44E-02	1.70E-01	7.23E-01
		AA	0.05	-2.39 (2.33)	3.05E-01	3040	0.04	-0.14 (1.71)	9.33E-01	4154	5.00E-01	5.89E-01	4.38E-01
		AI	0.04	-0.47 (2.17)	8.28E-01	2615	0.04	2.63 (1.90)	1.65E-01	4081	3.67E-01	3.73E-01	2.82E-01
		MA/H	0.07	-0.81 (3.01)	7.88E-01	922	0.08	1.19 (1.35)	3.78E-01	863	4.88E-01	6.54E-01	5.44E-01
rs2197089 *LPL*	T	EA	0.55	0.62 (0.54)	2.48E-01	8095	0.56	-0.86 (0.41)	3.54E-02	12431	3.32E-01	5.62E-02	2.82E-02
		AA	0.78	-0.63 (1.11)	5.68E-01	3049	0.78	0.41 (0.76)	5.87E-01	5560	9.00E-01	7.33E-01	4.37E-01
		AI	0.41	0.24 (0.92)	7.97E-01	2617	0.41	1.77 (0.66)	6.99E-03	4208	1.91E-02	2.54E-02	1.73E-01
		MA/H	0.46	0.64 (1.61)	6.92E-01	928	0.48	-0.21 (0.71)	7.70E-01	1599	9.14E-01	8.86E-01	6.30E-01
rs328 *LPL*	C	EA	0.90	-0.96 (0.90)	2.89E-01	7900	0.90	0.85 (0.69)	2.17E-01	12172	7.32E-01	2.66E-01	1.11E-01
		AA	0.93	-1.12 (2.28)	6.21E-01	2487	0.93	-1.37 (1.35)	3.12E-01	4765	2.62E-01	5.31E-01	9.27E-01
		AI	0.97	-8.21 (2.76)	2.98E-03	2620	0.97	-4.89 (2.24)	2.92E-02	4216	3.66E-04	1.12E-03	3.51E-01
		MA/H	0.94	-7.68 (3.34)	2.17E-02	921	0.93	-0.48 (1.51)	7.51E-01	1593	2.17E-01	6.81E-02	4.99E-02
rs6586891 *LPL*	A	EA	0.65	0.66 (0.60)	2.71E-01	6881	0.65	-0.09 (0.55)	8.68E-01	10926	5.35E-01	5.39E-01	3.56E-01
		AA	0.84	-0.02 (1.39)	9.87E-01	2512	0.84	1.72 (1.05)	1.03E-01	4872	1.97E-01	2.65E-01	3.18E-01
		AI	0.45	-0.92 (0.89)	3.00E-01	2607	0.45	0.72 (0.65)	2.65E-01	4201	7.73E-01	3.14E-01	1.35E-01
		MA/H	0.55	0.57 (3.70)	8.78E-01	182	0.58	0.16 (1.90)	9.33E-01	809	8.85E-01	9.85E-01	9.21E-01
rs6102059 *MAFB*	T	EA	0.30	-0.63 (0.83)	4.49E-01	4343	0.30	0.42 (0.85)	6.18E-01	4901	8.47E-01	6.63E-01	3.76E-01
		AA	0.41	-2.71 (1.63)	9.77E-02	1402	0.42	0.07 (1.47)	9.61E-01	1822	2.83E-01	2.54E-01	2.07E-01
		AI	--	--	--	--	--	--	--	--	--	--	--
		MA/H	0.26	-0.96 (4.55)	8.32E-01	181	0.30	-10.77 (6.80)	1.13E-01	78	2.91E-01	2.79E-01	2.31E-01
rs17145738 *MLXIPL*	T	EA	0.12	-0.06 (0.81)	9.39E-01	8103	0.12	1.24 (0.63)	5.02E-02	12435	1.36E-01	1.46E-01	2.04E-01
		AA	0.09	-1.65 (1.64)	3.13E-01	3050	0.09	2.93 (1.09)	7.40E-03	5551	9.51E-02	1.66E-02	2.00E-02
		AI	0.07	2.14 (1.65)	1.95E-01	2617	0.07	0.89 (1.27)	4.83E-01	4202	1.78E-01	3.38E-01	5.48E-01
		MA/H	0.08	-4.45 (3.03)	1.41E-01	929	0.07	-1.13 (1.37)	4.10E-01	1602	1.74E-01	2.41E-01	3.17E-01
rs2338104 *MMAB-MVK*	C	EA	0.46	-0.40 (0.52)	4.38E-01	8092	0.46	-0.93 (0.43)	2.86E-02	9558	2.88E-02	6.74E-02	4.33E-01
		AA	0.28	0.18 (1.03)	8.63E-01	3036	0.27	-1.48 (0.76)	5.00E-02	4156	1.40E-01	1.44E-01	1.93E-01
		AI	0.57	1.46 (0.90)	1.03E-01	2600	0.58	-1.02 (0.68)	1.31E-01	4045	8.26E-01	8.48E-02	2.71E-02
		MA/H	0.53	-0.21 (1.59)	8.95E-01	924	0.52	0.04 (0.84)	9.67E-01	870	9.81E-01	9.91E-01	8.92E-01
rs11206510 *PCSK9*	T	EA	0.81	3.37 (0.67)	5.35E-07	8103	0.81	0.84 (0.52)	1.09E-01	12436	1.40E-05	9.49E-07	2.95E-03
		AA	0.86	-2.03 (1.37)	1.40E-01	3043	0.86	1.09 (0.90)	2.25E-01	5560	8.38E-01	1.62E-01	5.77E-02
		AI	0.94	-0.10 (1.98)	9.59E-01	2620	0.94	-1.44 (1.50)	3.37E-01	4212	4.26E-01	6.30E-01	5.89E-01
		MA/H	0.90	2.34 (2.67)	3.80E-01	928	0.87	1.56 (1.04)	1.33E-01	1602	8.58E-02	2.20E-01	7.85E-01

rs11591147	T	EA	0.02	-18.91 (2.10)	2.41E-19	8573	0.02	-13.76 (1.74)	3.05E-15	13066	3.29E-32	7.59E-32	5.90E-02
PCSK9		AA	<0.01	-18.81 (9.12)	3.92E-02	2677	<0.01	-23.41 (6.00)	9.76E-05	5205	1.15E-05	5.97E-05	6.74E-01
		AI	0.01	-12.82 (8.52)	1.32E-01	1069	0.01	-13.13 (4.88)	7.16E-03	2337	2.06E-03	8.64E-03	9.75E-01
		MA/H	<0.01	-27.29 (13.89)	4.95E-02	743	0.01	-14.57 (4.40)	9.35E-04	1513	1.79E-04	6.05E-04	3.83E-01
rs7679	T	EA	0.82	-0.99 (0.86)	2.49E-01	5347	0.82	0.21 (0.86)	8.04E-01	6042	5.24E-01	5.00E-01	3.22E-01
PLTP		AA	0.96	-2.11 (2.44)	3.85E-01	2521	0.96	3.28 (2.11)	1.20E-01	3469	5.43E-01	2.05E-01	9.41E-02
		AI	0.95	-1.71 (1.92)	3.72E-01	2619	0.95	-1.11 (1.48)	4.55E-01	4080	2.56E-01	5.08E-01	8.04E-01
		MA/H	0.88	14.04 (5.98)	1.90E-02	183	0.92	-0.16 (10.60)	9.88E-01	81	4.19E-02	6.39E-02	2.44E-01
rs1501908	G	EA	0.63	1.63 (0.62)	9.02E-03	6567	0.64	0.67 (0.48)	1.62E-01	7546	6.87E-03	1.24E-02	2.25E-01
TIMD4		AA	0.37	1.26 (0.99)	2.03E-01	3044	0.37	0.42 (0.68)	5.37E-01	4158	2.18E-01	3.67E-01	4.86E-01
		AI	0.87	-3.19 (1.33)	1.63E-02	2603	0.88	-1.73 (1.06)	1.01E-01	4053	5.43E-03	1.45E-02	3.91E-01
		MA/H	0.76	0.82 (1.93)	6.72E-01	927	0.76	-1.33 (0.88)	1.29E-01	874	2.28E-01	2.89E-01	3.10E-01
rs2075650	A	EA	0.87	-5.28 (1.85)	4.31E-03	1266	0.88	-4.24 (1.65)	1.02E-02	1763	1.35E-04	6.25E-04	6.76E-01
TOMM40		AA	0.87	0.00 (3.25)	9.99E-01	570	0.86	-4.81 (3.49)	1.68E-01	433	3.48E-01	3.87E-01	3.14E-01
		AI	--	--	--	--	--	--	--	--	--	--	--
		MA/H	0.90	-9.57 (6.03)	1.12E-01	184	0.92	26.78 (10.15)	8.36E-03	81	9.85E-01	8.74E-03	2.09E-03
rs2954029	A	EA	0.54	1.98 (0.67)	3.02E-03	5571	0.53	2.16 (0.49)	1.09E-05	6520	1.16E-07	7.59E-07	8.29E-01
TRIB1		AA	0.67	-0.52 (2.42)	8.28E-01	524	0.68	-0.96 (1.06)	3.69E-01	693	3.63E-01	6.52E-01	8.70E-01
		AI	--	--	--	--	--	--	--	--	--	--	--
		MA/H	0.61	4.15 (1.81)	2.20E-02	746	0.63	0.58 (0.80)	4.69E-01	794	1.13E-01	5.57E-02	7.09E-02
rs471364	A	EA	0.89	1.96 (0.86)	2.24E-02	7633	0.88	0.37 (0.66)	5.78E-01	9178	6.62E-02	6.32E-02	1.43E-01
TTC39B		AA	0.81	0.57 (1.22)	6.39E-01	2961	0.81	-1.96 (0.85)	2.19E-02	4035	1.07E-01	6.47E-02	8.94E-02
		AI	0.97	-5.26 (2.57)	4.11E-02	2604	0.97	0.42 (2.05)	8.38E-01	4065	2.66E-01	1.22E-01	8.45E-02
		MA/H	0.92	1.50 (2.93)	6.09E-01	890	0.92	-0.15 (1.44)	9.18E-01	825	8.94E-01	8.73E-01	6.14E-01

[*]Abbreviations: RE=race/ethnicity: EA=European American; AA=African American; AI=American Indian; MA/H=Mexican American/Hispanic; CAF = coding allele frequency; SE=standard error.

[†]P-value for SNP-phenotype association in the sex-combined meta-analysis, not allowing for different effects by sex.

[‡]P-value for SNP-phenotype association in the sex-combined meta-analysis, allowing for different effects by sex.

[§]P-value for heterogeneity by sex.

[||]Data not available.

Supplementary Table 4. Sex-specific associations of GWAS-identified lipid SNPs with HDL levels in the PAGE study.

	Coded		Males				Females						
SNP/Gene	Allele	R/E[*]	CAF	Beta (SE)	P	N	CAF	Beta (SE)	P	N	P_{comb}[†]	P_{diff}[‡]	P_{het}[§]
rs1883025	A	EA	0.24	-1.00 (0.25)	7.28E-05	6226	0.25	-0.25 (0.24)	3.00E-01	7144	5.19E-04	2.22E-04	2.90E-02
ABCA1		AA	0.34	-0.54 (0.82)	5.07E-01	823	0.34	0.19 (0.39)	6.31E-01	1032	8.80E-01	7.15E-01	4.21E-01
		AI	--[||]	--	--	--	--	--	--	--	--	--	--
		MA/H	0.28	-0.38 (0.56)	4.95E-01	1148	0.26	-0.19 (0.32)	5.48E-01	1187	3.90E-01	6.62E-01	7.66E-01
rs3890182	A	EA	0.12	-0.98 (0.27)	3.33E-04	8761	0.12	-0.88 (0.25)	4.20E-04	14870	5.09E-07	3.14E-06	7.82E-01
ABCA1		AA	0.12	-0.76 (0.54)	1.63E-01	3348	0.12	-0.73 (0.37)	4.48E-02	6338	1.45E-02	5.04E-02	9.74E-01
		AI	0.04	-2.21 (1.09)	4.26E-02	2605	0.04	-0.44 (0.89)	6.24E-01	4194	9.67E-02	1.13E-01	2.07E-01
		MA/H	0.08	-0.18 (0.90)	8.45E-01	1319	0.09	-0.37 (0.49)	4.48E-01	2021	4.48E-01	7.36E-01	8.46E-01
rs4149268	A	EA	0.37	-0.53 (0.23)	1.93E-02	6214	0.37	-0.01 (0.21)	9.70E-01	6988	1.02E-01	6.45E-02	9.38E-02
ABCA1		AA	0.67	0.54 (0.49)	2.74E-01	2230	0.68	-0.29 (0.33)	3.90E-01	2873	9.26E-01	3.80E-01	1.65E-01
		AI	--	--	--	--	--	--	--	--	--	--	--
		MA/H	0.31	0.43 (0.51)	4.03E-01	1271	0.32	-0.39 (0.30)	1.93E-01	1207	4.85E-01	3.02E-01	1.67E-01
rs6544713	T	EA	0.31	-0.67 (0.24)	4.98E-03	6127	0.32	-0.13 (0.26)	6.07E-01	11515	1.55E-02	1.69E-02	1.30E-01
ABCG8		AA	0.17	0.32 (0.52)	5.45E-01	2821	0.17	-0.22 (0.39)	5.73E-01	5744	9.29E-01	7.10E-01	4.11E-01
		AI	0.09	-1.29 (0.71)	7.15E-02	2601	0.09	0.06 (0.60)	9.22E-01	4188	2.76E-01	1.96E-01	1.50E-01
		MA/H	0.16	-2.56 (1.51)	9.05E-02	182	0.22	-0.50 (0.88)	5.71E-01	851	1.79E-01	2.03E-01	2.40E-01
rs1748195	C	EA	0.67	-0.22 (0.24)	3.58E-01	6055	0.66	-0.10 (0.20)	6.19E-01	11538	8.34E-01	5.80E-01	3.06E-01
ANGPLT3		AA	0.34	0.44 (0.56)	4.31E-01	1879	0.35	0.08 (0.29)	7.77E-01	4614	9.08E-01	7.04E-01	4.07E-01
		AI	--	--	--	--	0.61	1.53 (2.12)	4.71E-01	132	4.71E-01	--	--
		MA/H	0.60	0.48 (0.52)	3.55E-01	1149	0.59	0.27 (0.27)	3.08E-01	1956	6.32E-01	3.88E-01	1.97E-01
rs2967605	A	EA	0.18	-0.67 (0.23)	3.51E-03	8757	0.18	-0.80 (0.23)	3.68E-04	10193	4.52E-06	2.46E-05	6.83E-01
ANGPTL4		AA	0.21	-0.96 (0.44)	2.89E-02	3352	0.21	-0.65 (0.31)	3.57E-02	4520	2.93E-03	1.01E-02	5.63E-01
		AI	0.30	-0.06 (0.44)	8.86E-01	2601	0.29	-0.51 (0.35)	1.46E-01	4052	2.21E-01	3.44E-01	4.25E-01
		MA/H	0.22	-1.08 (0.56)	5.41E-02	1326	0.24	-0.25 (0.32)	4.35E-01	1259	1.03E-01	1.15E-01	1.97E-01
rs28927680	G	EA	0.93	1.50 (0.34)	9.48E-06	9479	0.93	0.71 (0.32)	2.50E-02	15704	3.10E-06	4.40E-06	8.94E-02
APOA1/C3/		AA	0.84	-0.24 (0.47)	6.07E-01	3641	0.84	-0.18 (0.32)	5.69E-01	6798	4.47E-01	7.45E-01	9.15E-01
A4/A5		AI	0.87	1.08 (0.62)	8.06E-02	2604	0.87	1.00 (0.43)	2.06E-02	4195	3.75E-03	1.48E-02	9.16E-01
		MA/H	0.85	-0.34 (0.67)	6.17E-01	1328	0.86	-0.53 (0.37)	1.54E-01	2038	1.36E-01	3.20E-01	8.02E-01
rs3135506	C	EA	0.06	-1.65 (0.37)	7.70E-06	8521	0.06	-1.53 (0.37)	4.27E-05	9925	1.38E-09	1.02E-08	8.29E-01
APOA5		AA	0.06	-1.82 (0.87)	3.59E-02	2808	0.06	-1.29 (0.59)	2.74E-02	3776	2.68E-03	9.69E-03	6.14E-01
		AI	0.12	-1.41 (0.62)	2.23E-02	2613	0.13	-1.09 (0.44)	1.29E-02	4065	8.11E-04	3.33E-03	6.68E-01

		MA/H	0.14	-1.38 (0.70)	4.80E-02	1320	0.13	-0.42 (0.40)	3.01E-01	1250	5.92E-02	8.30E-02	2.34E-01
rs964184 *APOA1/C3/ A4/A5*	C	EA	0.86	1.85 (0.39)	1.88E-06	4180	0.86	1.71 (0.42)	3.78E-05	9351	3.08E-10	2.35E-09	8.15E-01
		AA	0.80	-1.55 (0.89)	8.22E-02	1033	0.80	0.90 (0.57)	1.15E-01	3541	6.99E-01	6.37E-02	2.07E-02
		AI	--	--	--	--	0.78	1.32 (2.48)	5.96E-01	132	5.96E-01	--	--
		MA/H	--	--	--	--	0.75	3.17 (0.89)	3.44E-04	772	3.44E-04	--	--
rs562338 *APOB*	T	EA	0.19	0.06 (0.25)	8.11E-01	6978	0.19	0.14 (0.28)	6.24E-01	12803	6.13E-01	8.62E-01	8.39E-01
		AA	0.59	0.90 (0.41)	2.84E-02	2524	0.59	0.46 (0.32)	1.42E-01	5298	1.23E-02	3.07E-02	4.01E-01
		AI	0.08	-0.09 (0.72)	8.99E-01	2608	0.08	0.51 (0.58)	3.80E-01	4191	5.47E-01	6.75E-01	5.15E-01
		MA/H	0.16	-0.67 (1.61)	6.76E-01	182	0.09	1.89 (1.03)	6.54E-02	853	1.84E-01	1.68E-01	1.80E-01
rs693 *APOB*	T	EA	0.49	-0.47 (0.20)	2.01E-02	6531	0.49	-0.37 (0.22)	9.36E-02	12409	4.43E-03	1.65E-02	7.44E-01
		AA	0.24	-0.09 (0.49)	8.55E-01	2460	0.23	-0.74 (0.37)	4.32E-02	5190	8.36E-02	1.27E-01	2.88E-01
		AI	0.33	-0.20 (0.43)	6.35E-01	2606	0.33	-0.66 (0.32)	3.86E-02	4182	5.22E-02	1.05E-01	3.92E-01
		MA/H	0.37	-0.36 (1.26)	7.75E-01	182	0.41	-0.62 (0.86)	4.70E-01	852	4.49E-01	7.39E-01	8.62E-01
rs754523 *APOB*	T	EA	0.68	0.32 (0.22)	1.43E-01	6534	0.68	0.04 (0.27)	8.94E-01	7766	2.25E-01	3.40E-01	4.07E-01
		AA	0.78	0.48 (0.50)	3.34E-01	2454	0.79	-0.07 (0.44)	8.74E-01	3353	6.01E-01	6.19E-01	4.08E-01
		AI	0.69	0.28 (0.44)	5.23E-01	2604	0.68	0.57 (0.33)	8.99E-02	4050	8.21E-02	1.94E-01	6.10E-01
		MA/H	0.71	2.29 (1.23)	6.17E-02	180	0.69	5.67 (2.46)	2.11E-02	80	6.92E-03	1.22E-02	2.19E-01
rs10402271 *APOE/C1/C 4*	T	EA	0.67	0.66 (0.24)	6.54E-03	5543	0.67	-0.004 (0.29)	9.89E-01	6630	3.48E-02	2.47E-02	8.62E-02
		AA	0.84	0.65 (0.72)	3.64E-01	1619	0.84	0.28 (0.70)	6.90E-01	2176	3.57E-01	6.12E-01	7.12E-01
		AI	--	--	--	--	--	--	--	--	--	--	--
		MA/H	0.61	0.64 (1.31)	6.28E-01	183	0.63	0.88 (2.27)	7.00E-01	81	5.40E-01	8.25E-01	9.27E-01
rs4420638 *APOE/C1/C 4*	A	EA	0.82	1.25 (0.26)	1.72E-06	7593	0.83	0.67 (0.23)	3.61E-03	13605	9.13E-08	1.53E-07	9.43E-02
		AA	0.79	-1.62 (0.59)	6.37E-03	2150	0.80	-0.59 (0.34)	8.57E-02	5096	4.39E-03	5.52E-03	1.31E-01
		AI	0.92	0.97 (0.72)	1.77E-01	2611	0.92	1.35 (0.60)	2.39E-02	4189	9.29E-03	3.13E-02	6.88E-01
		MA/H	0.90	1.80 (0.88)	4.02E-02	1094	0.89	0.60 (0.45)	1.88E-01	1898	3.48E-02	5.12E-02	2.23E-01
rs4803750 *BCL3*	A	EA	0.93	-0.08 (0.46)	8.64E-01	5565	0.93	-1.16 (0.56)	3.77E-02	6649	1.48E-01	1.14E-01	1.33E-01
		AA	0.92	-1.47 (1.01)	1.46E-01	1623	0.92	1.28 (0.93)	1.67E-01	2193	9.73E-01	1.34E-01	4.50E-02
		AI	--	--	--	--	--	--	--	--	--	--	--
		MA/H	0.86	1.53 (1.78)	3.90E-01	183	0.84	-4.86 (3.34)	1.45E-01	81	9.41E-01	2.39E-01	9.11E-02
rs646776 *CELSR2/PSC R1/SORT1*	A	EA	0.78	-0.66 (0.25)	6.98E-03	6702	0.79	-0.25 (0.29)	3.98E-01	7978	9.13E-03	1.83E-02	2.74E-01
		AA	0.64	-0.68 (0.42)	1.06E-01	2750	0.65	-0.24 (0.37)	5.05E-01	3799	1.18E-01	2.17E-01	4.36E-01
		AI	0.80	-1.15 (0.70)	1.02E-01	1078	0.80	-0.74 (0.55)	1.79E-01	1747	3.89E-02	1.07E-01	6.48E-01
		MA/H	0.80	2.29 (1.57)	1.43E-01	182	0.74	0.69 (2.72)	7.99E-01	80	1.63E-01	3.31E-01	6.09E-01
rs599839 *CELSR2/PSC*	A	EA	0.77	-0.68 (0.24)	4.30E-03	6986	0.77	-0.08 (0.28)	7.87E-01	8150	1.84E-02	1.63E-02	1.02E-01
		AA	0.29	-0.83 (0.45)	6.29E-02	2524	0.28	0.30 (0.40)	4.55E-01	3471	4.97E-01	1.34E-01	5.93E-02

R1/SORT1		AI	0.78	-0.86 (0.47)	6.82E-02	2585	0.78	-0.64 (0.38)	9.10E-02	4043	1.39E-02	4.54E-02	7.21E-01
		MA/H	0.77	1.59 (1.52)	2.95E-01	178	0.72	-0.33 (2.62)	9.00E-01	81	3.99E-01	5.73E-01	5.26E-01
rs12596776	C	EA	0.90	-1.22 (0.38)	1.26E-03	6202	0.90	-0.96 (0.34)	5.35E-03	7000	2.34E-05	1.13E-04	6.12E-01
CETP		AA	0.94	0.61 (0.98)	5.35E-01	2234	0.94	-1.39 (0.66)	3.41E-02	2859	1.57E-01	8.74E-02	9.03E-02
		AI	--	--	--	--	--	--	--	--	--	--	--
		MA/H	0.94	0.43 (0.94)	6.50E-01	1314	0.94	-0.56 (0.57)	3.25E-01	1265	5.45E-01	5.56E-01	3.69E-01
rs1566439	A	EA	0.60	-0.65 (0.20)	1.43E-03	7335	0.60	-0.29 (0.19)	1.22E-01	8725	9.45E-04	1.87E-03	2.03E-01
CETP		AA	0.78	0.59 (0.53)	2.66E-01	2443	0.78	-0.05 (0.37)	8.88E-01	3223	6.02E-01	5.34E-01	3.22E-01
		AI	--	--	--	--	--	--	--	--	--	--	--
		MA/H	0.54	-0.83 (0.48)	8.05E-02	1321	0.54	0.02 (0.27)	9.45E-01	1267	4.16E-01	2.16E-01	1.21E-01
rs1864163	A	EA	0.24	-2.57 (1.55)	9.67E-02	182	0.21	-1.16 (2.68)	6.66E-01	109	9.80E-02	2.29E-01	6.49E-01
CETP		AA	0.27	-2.95 (1.11)	8.13E-03	367	0.24	-2.08 (2.55)	4.15E-01	84	5.93E-03	2.16E-02	7.54E-01
		AI	--	--	--	--	--	--	--	--	--	--	--
		MA/H	0.27	-2.08 (1.32)	1.15E-01	183	0.30	-5.43 (2.71)	4.48E-02	82	2.18E-02	3.87E-02	2.66E-01
rs3764261	T	EA	0.32	3.02 (0.19)	8.65E-58	8926	0.33	3.20 (0.18)	1.16E-68	10552	1.76E-124	3.53E-123	4.86E-01
CETP		AA	0.31	3.11 (0.39)	6.85E-16	3538	0.32	1.93 (0.27)	1.60E-12	4788	1.73E-25	9.32E-26	1.24E-02
		AI	0.31	2.82 (0.43)	5.01E-11	2589	0.31	2.85 (0.33)	5.62E-18	4036	1.88E-27	2.38E-26	9.49E-01
		MA/H	0.34	2.21 (0.52)	2.11E-05	1272	0.33	1.69 (0.29)	9.89E-09	1207	1.39E-12	8.33E-12	3.86E-01
rs9989419	A	EA	0.40	-1.83 (0.20)	8.08E-21	7699	0.40	-2.47 (0.23)	2.23E-26	8954	1.38E-44	2.52E-44	3.71E-02
CETP		AA	0.60	0.55 (0.40)	1.68E-01	2809	0.60	-0.58 (0.35)	9.48E-02	3902	7.22E-01	9.59E-02	3.27E-02
		AI	0.26	-1.99 (0.45)	1.02E-05	2601	0.25	-1.63 (0.37)	8.38E-06	4049	4.40E-10	2.82E-09	5.34E-01
		MA/H	0.34	-1.95 (1.22)	1.09E-01	181	0.40	-4.50 (2.45)	6.63E-02	81	2.43E-02	5.13E-02	3.51E-01
rs16996148	T	EA	0.08	0.17 (0.32)	5.93E-01	8720	0.08	-0.09 (0.30)	7.64E-01	14809	8.83E-01	8.29E-01	5.52E-01
CILP2/PBX4		AA	0.15	-0.24 (0.50)	6.37E-01	3325	0.15	-0.50 (0.33)	1.32E-01	6315	1.28E-01	2.87E-01	6.69E-01
/NCAN1		AI	0.04	-1.79 (1.02)	7.79E-02	2610	0.04	-0.80 (0.78)	3.03E-01	4195	5.86E-02	1.24E-01	4.41E-01
		MA/H	0.06	0.54 (1.01)	5.93E-01	1328	0.06	0.69 (0.58)	2.36E-01	2041	1.96E-01	4.29E-01	8.98E-01
rs174547	T	EA	0.66	0.82 (0.22)	1.52E-04	7140	0.66	0.60 (0.20)	2.86E-03	8055	1.88E-06	8.88E-06	4.70E-01
FADS1		AA	0.81	0.99 (0.62)	1.09E-01	3323	0.92	0.65 (0.46)	1.59E-01	4472	3.71E-02	1.03E-01	6.54E-01
		AI	0.17	0.33 (0.58)	5.70E-01	2202	0.15	0.59 (0.52)	2.50E-01	3492	2.17E-01	4.40E-01	7.30E-01
		MA/H	0.38	0.43 (0.49)	3.79E-01	1269	0.40	0.89 (0.28)	1.32E-03	1211	1.25E-03	3.90E-03	4.12E-01
rs2144300	T	EA	0.60	0.21 (0.19)	2.66E-01	8671	0.60	0.63 (0.17)	2.12E-04	14688	5.04E-04	5.62E-04	9.10E-02
GALNT2		AA	0.14	0.71 (0.52)	1.69E-01	3337	0.16	0.10 (0.32)	7.54E-01	6342	3.22E-01	3.70E-01	3.15E-01
		AI	0.54	0.59 (0.40)	1.38E-01	2599	0.55	0.02 (0.31)	9.58E-01	4180	3.41E-01	3.33E-01	2.56E-01
		MA/H	0.57	-0.15 (0.49)	7.56E-01	1332	0.55	0.41 (0.26)	1.14E-01	2039	2.10E-01	2.72E-01	3.10E-01
rs1260326	T	EA	0.42	-0.01 (0.20)	9.70E-01	7312	0.42	0.19 (0.18)	2.85E-01	13468	4.38E-01	5.64E-01	4.60E-01

GCKR		AA	0.17	1.00 (0.92)	2.80E-01	1019	0.15	0.56 (0.43)	1.89E-01	3203	9.96E-02	2.35E-01	6.67E-01
		AI	--	--	--	--	0.29	0.20 (2.04)	9.22E-01	131	9.22E-01	--	--
		MA/H	0.32	0.10 (0.54)	8.47E-01	1135	0.33	0.42 (0.28)	1.35E-01	1942	1.58E-01	3.22E-01	5.98E-01
rs780094 *GCKR*	A	EA	0.40	-0.11 (0.21)	5.94E-01	6537	0.40	-0.02 (0.25)	9.52E-01	7775	6.52E-01	8.66E-01	7.70E-01
		AA	0.19	-0.06 (0.53)	9.11E-01	2458	0.18	0.76 (0.48)	1.11E-01	3359	2.67E-01	2.80E-01	2.51E-01
		AI	0.21	-0.75 (0.49)	1.31E-01	2607	0.20	-0.02 (0.42)	9.60E-01	4061	3.13E-01	3.20E-01	2.61E-01
		MA/H	0.34	-0.42 (1.38)	7.58E-01	182	0.33	2.00 (2.67)	4.53E-01	81	9.43E-01	7.20E-01	4.19E-01
rs12654264 *HMGCR*	A	EA	0.62	-0.02 (0.19)	9.00E-01	8756	0.62	-0.21 (0.17)	2.22E-01	14863	3.26E-01	4.71E-01	4.61E-01
		AA	0.67	-0.24 (0.37)	5.20E-01	3343	0.67	-0.11 (0.26)	6.59E-01	6328	4.66E-01	7.37E-01	7.79E-01
		AI	0.57	0.46 (0.42)	2.64E-01	2603	0.59	-0.01 (0.32)	9.65E-01	4194	5.17E-01	5.35E-01	3.62E-01
		MA/H	0.61	0.06 (0.51)	9.02E-01	1319	0.62	0.38 (0.27)	1.58E-01	2018	1.93E-01	3.67E-01	5.76E-01
rs2650000 *HNF1A*	T	EA	0.35	0.29 (0.19)	1.34E-01	8275	0.35	-0.08 (0.19)	6.61E-01	9796	4.66E-01	2.95E-01	1.67E-01
		AA	0.12	-0.36 (0.56)	5.26E-01	3271	0.12	0.75 (0.40)	5.75E-02	4397	2.36E-01	1.35E-01	1.07E-01
		AI	0.41	1.00 (0.40)	1.38E-02	2603	0.41	0.12 (0.31)	6.93E-01	4062	6.92E-02	4.47E-02	8.77E-02
		MA/H	0.37	0.7 (0.50)	1.59E-01	1327	0.36	0.46 (0.29)	1.12E-01	1260	3.75E-02	1.05E-01	6.78E-01
rs1800961 *HNF4A*	T	EA	0.03	-0.58 (0.53)	2.80E-01	8259	0.03	-1.33 (0.50)	8.10E-03	9790	7.63E-03	1.67E-02	3.02E-01
		AA	0.01	-7.68 (1.61)	1.99E-06	3267	0.01	0.60 (1.59)	7.05E-01	4395	2.15E-03	1.13E-05	2.59E-04
		AI	0.03	-1.24 (1.09)	2.56E-01	2609	0.03	-2.09 (0.91)	2.23E-02	4064	1.31E-02	3.84E-02	5.51E-01
		MA/H	0.04	-1.76 (1.19)	1.38E-01	1275	0.04	-1.30 (0.73)	7.43E-02	1211	2.17E-02	6.77E-02	7.38E-01
rs2271293 *LCAT*	A	EA	0.12	1.34 (0.29)	3.99E-06	8281	0.12	1.23 (0.26)	2.46E-06	14455	4.49E-11	3.52E-10	7.81E-01
		AA	0.07	1.77 (0.72)	1.41E-02	3268	0.08	0.49 (0.45)	2.77E-01	6224	2.64E-02	2.72E-02	1.31E-01
		AI	0.25	1.86 (0.45)	3.73E-05	2600	0.24	0.84 (0.36)	2.06E-02	4192	1.15E-05	1.38E-05	7.76E-02
		MA/H	0.12	0.97 (0.76)	2.00E-01	1270	0.15	0.24 (0.38)	5.31E-01	1979	2.55E-01	3.61E-01	3.89E-01
rs2228671 *LDLR*	T	EA	0.12	-0.17 (0.45)	7.10E-01	3040	0.12	0.22 (0.35)	5.24E-01	3848	7.83E-01	7.62E-01	4.94E-01
		AA	0.04	1.06 (1.44)	4.60E-01	1370	0.04	1.2 (0.84)	1.57E-01	1456	1.11E-01	2.79E-01	9.36E-01
		AI	--	--	--	--	--	--	--	--	--	--	--
		MA/H	0.08	0.48 (0.88)	5.90E-01	1310	0.08	-0.28 (0.52)	5.85E-01	1257	8.43E-01	7.45E-01	4.59E-01
rs6511720 *LDLR*	T	EA	0.12	-0.01 (0.29)	9.61E-01	7700	0.12	1.28 (0.32)	8.57E-05	13621	9.90E-03	4.42E-04	3.04E-03
		AA	0.14	-0.03 (0.58)	9.58E-01	2814	0.13	-0.06 (0.44)	8.83E-01	5732	8.81E-01	9.88E-01	9.64E-01
		AI	0.05	-0.05 (0.94)	9.60E-01	2598	0.04	1.55 (0.76)	4.24E-02	4191	1.22E-01	1.27E-01	1.88E-01
		MA/H	0.10	-1.27 (1.88)	5.01E-01	182	0.09	3.58 (1.37)	9.24E-03	852	8.77E-02	2.69E-02	3.77E-02
rs261332 *LIPC*	A	EA	0.20	1.77 (0.32)	3.91E-08	4439	0.2	1.79 (0.42)	2.00E-05	4926	3.52E-12	3.02E-11	9.64E-01
		AA	0.24	0.25 (0.67)	7.08E-01	1399	0.25	0.86 (0.66)	1.90E-01	1827	2.32E-01	3.95E-01	5.14E-01
		AI	--	--	--	--	--	--	--	--	--	--	--
		MA/H	0.20	-0.15 (1.49)	9.19E-01	179	0.16	1.03 (3.22)	7.49E-01	80	9.66E-01	9.45E-01	7.39E-01

rs4775041	C	EA	0.29	1.33 (0.20)	1.81E-11	8733	0.29	1.05 (0.19)	4.53E-08	10175	7.69E-18	4.70E-17	3.05E-01
LIPC		AA	0.14	0.2 (0.53)	7.09E-01	3344	0.13	0.81 (0.38)	3.26E-02	4498	5.05E-02	9.51E-02	3.49E-01
		AI	0.20	1.19 (0.48)	1.30E-02	2610	0.21	1.42 (0.39)	2.36E-04	4063	9.84E-06	5.25E-05	7.07E-01
		MA/H	0.18	1.72 (0.61)	4.79E-03	1328	0.18	0.38 (0.35)	2.75E-01	1258	1.85E-02	1.03E-02	5.78E-02
rs2156552	T	EA	0.17	-0.38 (0.24)	1.08E-01	8761	0.16	-0.56 (0.24)	1.80E-02	10208	4.90E-03	1.67E-02	6.04E-01
LIPG		AA	0.05	-1.22 (0.93)	1.90E-01	3349	0.04	0.26 (0.65)	6.95E-01	4510	6.67E-01	3.92E-01	1.94E-01
		AI	0.04	-1.40 (0.94)	1.39E-01	2605	0.04	-1.06 (0.93)	2.57E-01	4058	6.49E-02	1.76E-01	8.00E-01
		MA/H	0.07	0.68 (0.92)	4.60E-01	1319	0.08	0.55 (0.48)	2.53E-01	1256	1.75E-01	3.96E-01	9.05E-01
rs2197089	T	EA	0.55	0.61 (0.18)	6.44E-04	8769	0.55	0.83 (0.17)	5.35E-07	14869	1.99E-09	1.01E-08	3.76E-01
LPL		AA	0.78	0.81 (0.43)	5.90E-02	3362	0.78	0.80 (0.29)	5.97E-03	6351	8.55E-04	3.83E-03	9.88E-01
		AI	0.41	1.11 (0.41)	7.14E-03	2607	0.41	0.80 (0.31)	9.83E-03	4192	2.34E-04	9.53E-04	5.50E-01
		MA/H	0.46	1.39 (0.48)	3.54E-03	1330	0.48	0.48 (0.26)	6.09E-02	2032	2.43E-03	2.45E-03	9.31E-02
rs328	C	EA	0.90	-1.76 (0.31)	1.47E-08	8589	0.90	-1.93 (0.28)	3.34E-12	14628	3.04E-19	3.01E-18	6.84E-01
LPL		AA	0.93	-1.74 (0.85)	4.13E-02	2794	0.93	-1.19 (0.53)	2.50E-02	5552	2.90E-03	1.01E-02	5.78E-01
		AI	0.97	-0.49 (1.21)	6.89E-01	2610	0.97	-1.71 (1.08)	1.14E-01	4200	1.49E-01	2.65E-01	4.52E-01
		MA/H	0.94	-2.55 (1.00)	1.09E-02	1321	0.93	-1.07 (0.54)	4.85E-02	2022	3.20E-03	5.58E-03	1.95E-01
rs6586891	A	EA	0.65	0.99 (0.21)	2.27E-06	7001	0.65	0.90 (0.23)	9.82E-05	12798	9.45E-10	6.94E-09	7.80E-01
LPL		AA	0.84	0.60 (0.56)	2.77E-01	2522	0.84	0.30 (0.41)	4.75E-01	5319	2.22E-01	4.29E-01	6.56E-01
		AI	0.45	0.55 (0.41)	1.72E-01	2597	0.45	0.49 (0.31)	1.11E-01	4185	3.61E-02	1.10E-01	9.04E-01
		MA/H	0.55	-0.76 (1.17)	5.15E-01	183	0.58	1.48 (0.81)	6.63E-02	852	2.53E-01	1.50E-01	1.15E-01
rs6102059	T	EA	0.30	0.08 (0.29)	7.84E-01	4442	0.30	-0.05 (0.37)	8.88E-01	4951	8.97E-01	9.54E-01	7.80E-01
MAFB		AA	0.41	1.24 (0.61)	4.07E-02	1412	0.42	-0.14 (0.58)	8.07E-01	1832	2.16E-01	1.20E-01	9.92E-02
		AI	--	--	--	--	--	--	--	--	--	--	--
		MA/H	0.26	1.81 (1.46)	2.15E-01	182	0.30	-2.30 (2.61)	3.78E-01	80	5.15E-01	3.15E-01	1.69E-01
rs17145738	T	EA	0.12	0.81 (0.28)	3.50E-03	8777	0.12	0.91 (0.26)	3.64E-04	14877	4.25E-06	2.42E-05	7.79E-01
MLXIPL		AA	0.09	-0.53 (0.63)	3.99E-01	3361	0.09	-0.03 (0.42)	9.52E-01	6344	6.05E-01	7.00E-01	5.04E-01
		AI	0.07	0.58 (0.77)	4.55E-01	2607	0.07	0.19 (0.59)	7.43E-01	4186	4.76E-01	7.17E-01	6.91E-01
		MA/H	0.08	0.39 (0.91)	6.68E-01	1332	0.07	0.18 (0.52)	7.26E-01	2037	6.05E-01	8.58E-01	8.45E-01
rs2338104	C	EA	0.46	-0.34 (0.18)	5.58E-02	8767	0.46	-0.43 (0.18)	1.52E-02	10213	2.15E-03	8.44E-03	7.22E-01
MMAB-		AA	0.28	-0.67 (0.40)	9.38E-02	3349	0.26	-0.16 (0.29)	5.76E-01	4512	1.49E-01	2.10E-01	3.08E-01
MVK		AI	0.57	-0.46 (0.40)	2.55E-01	2590	0.58	0.23 (0.32)	4.69E-01	4022	8.89E-01	4.02E-01	1.80E-01
		MA/H	0.53	-0.74 (0.47)	1.18E-01	1327	0.52	-0.44 (0.29)	1.28E-01	1265	3.45E-02	9.25E-02	5.89E-01
rs11206510	T	EA	0.81	0.27 (0.23)	2.37E-01	8776	0.81	0.02 (0.21)	9.21E-01	14877	3.85E-01	4.94E-01	4.18E-01
PCSK9		AA	0.86	-0.02 (0.52)	9.70E-01	3355	0.86	-0.53 (0.35)	1.31E-01	6355	2.02E-01	3.19E-01	4.19E-01
		AI	0.94	0.21 (0.87)	8.05E-01	2610	0.94	-1.51 (0.73)	3.82E-02	4196	1.54E-01	1.13E-01	1.27E-01

		MA/H	0.90	0.54 (0.77)	4.84E-01	1332	0.87	0.54 (0.38)	1.59E-01	2037	1.16E-01	2.90E-01	9.96E-01
rs11591147	T	EA	0.01	0.42 (0.73)	5.62E-01	9252	0.02	0.18 (0.71)	8.00E-01	15503	5.58E-01	8.18E-01	8.10E-01
PCSK9		AA	<0.01	1.59 (3.81)	6.77E-01	2978	<0.01	2.38 (2.07)	2.52E-01	6678	2.28E-01	4.76E-01	8.56E-01
		AI	0.01	-0.56 (3.55)	8.75E-01	1061	0.01	4.18 (2.38)	7.87E-02	2318	1.70E-01	2.11E-01	2.67E-01
		MA/H	<0.01	4.11 (3.93)	2.96E-01	1143	0.01	-2.09 (1.66)	2.07E-01	1942	4.50E-01	2.62E-01	1.46E-01
rs7679	T	EA	0.82	0.10 (0.30)	7.29E-01	5439	0.82	1.68 (0.37)	6.27E-06	6089	1.87E-03	3.45E-05	9.85E-04
PLTP		AA	0.96	0.79 (0.93)	3.96E-01	2531	0.96	0.63 (0.88)	4.76E-01	3481	2.71E-01	5.41E-01	8.99E-01
		AI	0.95	0.63 (0.84)	4.56E-01	2609	0.95	0.19 (0.73)	7.89E-01	4057	4.91E-01	7.31E-01	6.97E-01
		MA/H	0.88	1.31 (1.94)	4.98E-01	184	0.92	1.21 (4.09)	7.67E-01	83	4.60E-01	7.61E-01	9.82E-01
rs1501908	G	EA	0.63	0.09 (0.21)	6.69E-01	7211	0.63	-0.03 (0.20)	8.83E-01	8160	8.53E-01	9.03E-01	6.80E-01
TIMD4		AA	0.37	0.24 (0.38)	5.24E-01	3352	0.37	0.07 (0.26)	7.96E-01	4508	5.65E-01	7.90E-01	7.07E-01
		AI	0.87	0.28 (0.58)	6.32E-01	2593	0.88	0.16 (0.51)	7.57E-01	4030	5.84E-01	8.50E-01	8.77E-01
		MA/H	0.77	0.03 (0.57)	9.58E-01	1327	0.76	-0.21 (0.31)	5.03E-01	1269	5.75E-01	7.98E-01	7.10E-01
rs2075650	A	EA	0.87	-0.05 (0.72)	9.46E-01	1282	0.88	0.64 (0.76)	3.99E-01	1796	5.96E-01	6.99E-01	5.10E-01
TOMM40		AA	0.87	0.86 (1.16)	4.61E-01	570	0.86	0.61 (1.48)	6.78E-01	433	4.03E-01	6.99E-01	8.97E-01
		AI	--	--	--	--	--	--	--	--	--	--	--
		MA/H	0.9	0.01 (1.93)	9.94E-01	185	0.92	-0.97 (4.00)	8.08E-01	83	9.21E-01	9.71E-01	8.24E-01
rs2954029	A	EA	0.54	-0.27 (0.22)	2.22E-01	6227	0.53	-0.62 (0.20)	1.87E-03	7142	1.76E-03	3.75E-03	2.41E-01
TRIB1		AA	0.67	0.72 (0.80)	3.68E-01	824	0.68	-0.44 (0.39)	2.65E-01	1036	5.49E-01	3.59E-01	1.93E-01
		AI	--	--	--	--	--	--	--	--	--	--	--
		MA/H	0.61	-0.06 (0.53)	9.17E-01	1145	0.63	0.01 (0.28)	9.72E-01	1187	9.86E-01	9.94E-01	9.13E-01
rs471364	A	EA	0.89	0.86 (0.29)	2.88E-03	8282	0.88	-0.23 (0.28)	4.15E-01	9809	1.41E-01	8.42E-03	6.58E-03
TTC39B		AA	0.81	0.09 (0.47)	8.50E-01	3268	0.81	0.34 (0.34)	3.09E-01	4391	3.49E-01	5.86E-01	6.60E-01
		AI	0.97	3.19 (1.13)	4.62E-03	2594	0.97	-1.19 (0.99)	2.30E-01	4042	3.33E-01	8.79E-03	3.50E-03
		MA/H	0.92	0.15 (0.90)	8.65E-01	1277	0.92	-0.04 (0.51)	9.41E-01	1210	9.85E-01	9.83E-01	8.54E-01

[*]Abbreviations: RE=race/ethnicity: EA=European American; AA=African American; AI=American Indian; MA/H=Mexican American/Hispanic; CAF = coding allele frequency; SE=standard error.

[†]P-value for SNP-phenotype association in the sex-combined meta-analysis, not allowing for different effects by sex.

[‡]P-value for SNP-phenotype association in the sex-combined meta-analysis, allowing for different effects by sex.

[§]P-value for heterogeneity by sex.

[||]Data not available.

Supplementary Figure 1. Sample sizes required to detect interaction for SNPs with varying minor allele frequencies and effect sizes.

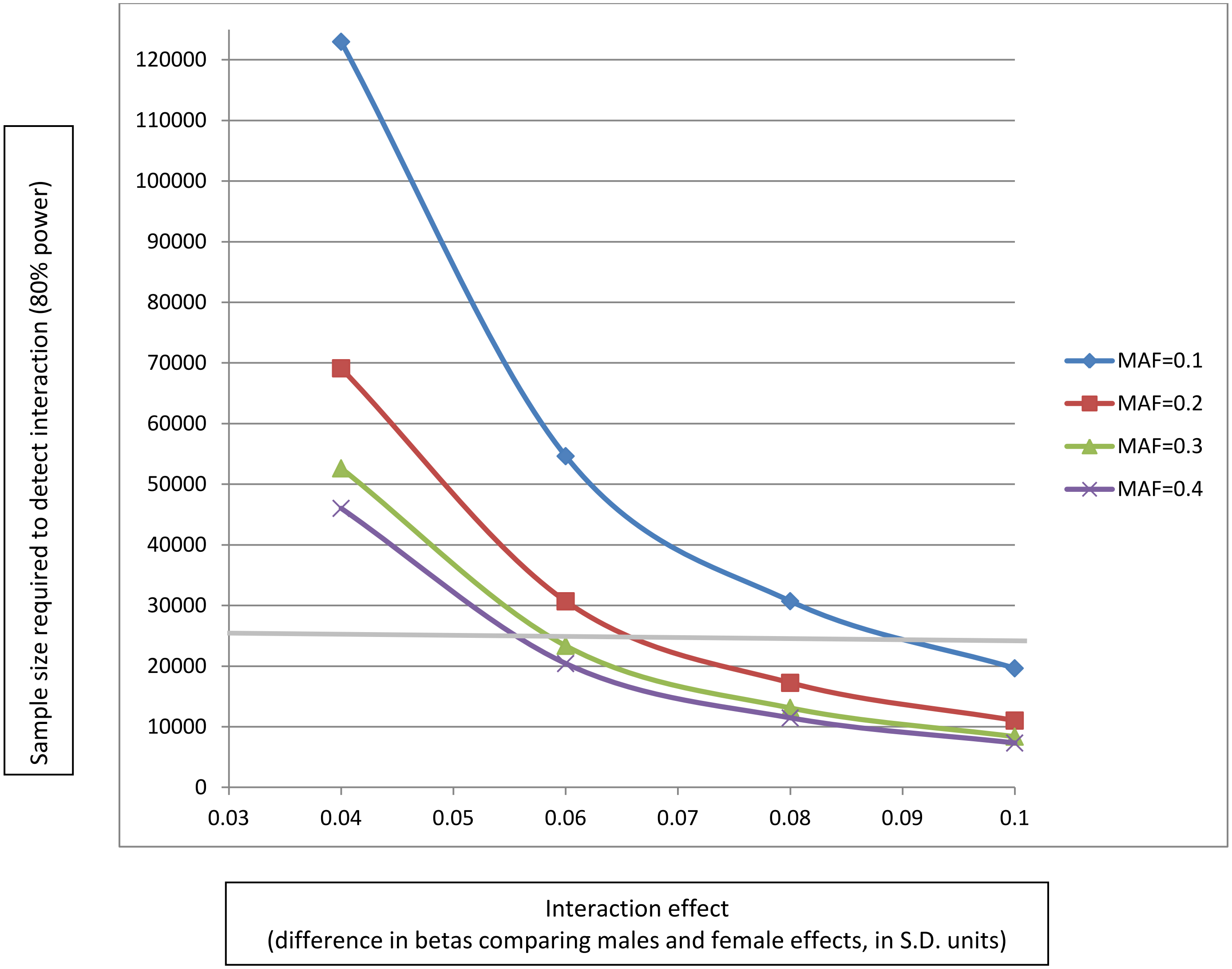

Supplement: Additional file 1 — This file contains Supplementary Tables 1-4 and Supplementary Figure 1 as described in the text. [file 1471-2156-14-33-S1.pdf]
